# Genome analysis to decipher syntrophy in the bacterial consortium ‘SCP’ for azo dye degradation

**DOI:** 10.1186/s12866-021-02236-9

**Published:** 2021-06-11

**Authors:** Sandhya Nanjani, Dhiraj Paul, Hareshkumar Keharia

**Affiliations:** 1 Post Graduate Department of Biosciences, UGC Centre of Advanced Study, Sardar Patel University, Satellite Campus, Vadtal Road, Bakrol, Anand, Gujarat 388 315 India; 2 Microbial Culture Collection, National Centre for Microbial Resource, National Centre for Cell Science, Savitribai Phule University of Pune Campus, Pune, India

**Keywords:** Dye degradation, Consortium, Azo-reductase, Genomics, Decolorization

## Abstract

**Background:**

A bacterial consortium SCP comprising three bacterial members, viz. *Stenotrophomonas acidaminiphila* APG1, *Pseudomonas stutzeri* APG2 and *Cellulomonas* sp. APG4 was developed for degradation of the mono-azo dye, Reactive Blue 28. The genomic analysis of each member of the SCP consortium was done to elucidate the catabolic potential and role of the individual organism in dye degradation.

**Results:**

The genes for glycerol utilization were detected in the genomes of APG2 and APG4, which corroborated with their ability to grow on a minimal medium containing glycerol as the sole co-substrate. The genes for azoreductase were identified in the genomes of APG2 and APG4, while no such trait could be determined in APG1. In addition to co-substrate oxidation and dye reduction, several other cellular functions like chemotaxis, signal transduction, stress-tolerance, repair mechanisms, aromatic degradation, and copper tolerance associated with dye degradation were also annotated. A model for azo dye degradation is postulated, representing the predominant role of APG4 and APG2 in dye metabolism while suggesting an accessory role of APG1.

**Conclusions:**

This exploratory study is the first-ever attempt to divulge the genetic basis of azo-dye co-metabolism by cross-genome comparisons and can be harnessed as an example for demonstrating microbial syntrophy.

**Supplementary Information:**

The online version contains supplementary material available at 10.1186/s12866-021-02236-9.

## Background

Reactive azo dyes are a class of refractory pollutants containing azo bond/s (−N_=_N-) linked to the skeleton of substituted or non-substituted aromatic rings [1]. These dye laden effluents predominantly from textile and dye-manufacturing units are discharged in the aquatic eco-system with consequent deleterious repercussions on the aquatic and terrestrial ecosystem [2]. Many azo dyes or their intermediates have been demonstrated to be mutagenic and/ or carcinogenic and hence have gained recognition as priority pollutants [3]. Thus, an effective treatment technology for remediation of such xenobiotics before their discharge in the eco-system is of prime concern [4]. The microbes have been characterized to cleave azo bond either by oxidative or reductive mechanisms [5]. Some organisms efficiently degrade azo dyes via ligninolytic mechanisms utilizing enzymes like laccases, manganese peroxidases, oxidases, etc. [6]. In contrast to this, the degradation under anaerobic or anoxic conditions commences with the breakage of the azo bridge (−N=N-), either by cytoplasmic azo-reductases or non-specifically by cellular components like that of the electron transport chain [1]. The dye reduction results in the appearance of complex intermediates, which are subsequently deaminated and/or desulphonated [7]. If the reduction occurs symmetrically, it results in the formation of aromatic amines, which can be further transformed by oxidation [8]. Moreover, the limitations in electron transfer can affect the rate of dye reduction, which is usually overcome by the introduction of redox mediators (RMs) in the system. RMs are small organic molecules, e.g., phenazine, cobalamin, riboflavin, quinone-derivatives, etc., capable of shuttling electrons in multiple redox reactions. These accelerate the decolorization by decreasing the activation energy of the entire reaction [9]. To achieve efficient decolorization, a neoteric approach involving use of microbial communities with or without redox mediators is gaining attention [10].

The basic approach to develop a synthetic consortium relies on the manipulation of cellular signaling, communication, antagonism, spatial organization and cooperation [11]. However, an alternative and simplistic manner of modeling a consortium would be to mix the key microbial candidates with the function of interest and establish cooperation. The selection of cultures should rely on the understanding of social behavior within the microbial cohorts and the impact of individuals on the functionality. The social intellect of the cultures in a community further depends on the metabolite-mediated cross-talks [12]. Many scientific groups have worked on the development of a consortium or mixed culture for the bioremediation of reactive azo dyes [13–15]. However, the complexities underlying the group of microorganisms during the bioremediation of dyes have not been well-understood. Furthermore, the reports describing the molecular mechanisms of dye degradation and identifying the genes involved in the entire process are very scarce. The genomic analysis can reveal the details of dye degradation potential and further open up a new vista in degradative pathways [16]. Table 1 provides the details of dye degraders isolated from various sources, including sewage treatment plants, hypersaline ponds and rhizosphere of plants, whose genome-mining was conducted to decipher the catabolism of the azo dyes [21, 23].

**Table 1:** Determinants of dye degradation obtained from whole genome sequencing of organisms isolated from various sources

Isolate and accession number	Source	Dye degradation	Proteins/genes for dye degradation identified from genome analysis	Reference
***Novibacillus thermophilus*** **SG-1** CP09699.1	Isolated from saline soil collected from Guangdong Province, China	Anaerobically decolorized azo dye, Orange I at high temperature	Riboflavin biosynthesis protein	[1]
***Bacillus endophyticus*** **2102** ALIM00000000	Isolated form a hypersaline pond located in South Korea	Not available	Several flavin dependent NADH azo-reductase, NADPH dependent azoreductase	[17]
***Enterococcus*** **sp. Strain C1** AKKS00000000	Isolated form a sewage oxidation pond in Malaysia	Microaerophlic decolorization of amaranth dye	(FMN)-dependent NADH azoreductase, copper amine oxidase, and sulfatase	[18]
***Citrobacter*** **sp. strain A1** AKTT01000000	Co-isolated with *Enterococcus* sp. Strain C1 form a sewage oxidation pond	Microaerophilic azo dye degradation followed by oxid-ative transformation of intermediates	Flavin reductase; Genes for deami- Nation and desulfonation	[7]
***Shewanella decolorationis*** **S12** AXZL00000000	Isolated from waste water treatment plant of a textile-printing industry in China	Variety of azo and anthroquinone dyes	None	[19, 20]
***Bacillus subtilis*** **C3** JYOG00000000	Isolated from a common effluent treatment plant (CETP)	Microaerophlic degradation of various dyes	Genes encoding for enzymes involved in azo reduction	[21]
***Serratia nematodiphila*** **MB307** MTBJ01000001– MTBJ01000031	Isolated from rhizo-sphere of *Cannabis sativa* growing in the effluent-contaminated soil in Pakistan	Degradation of sulphonated azo dyes viz., Methyl orange and Congo red	14 monooxygenases and 5 copies of dioxygenases	[22]
***Trichosporon akiyoshidainum*** **HP-2023** PQXP00000000.1	Isolated from the rhizosphere of *Cinnamomum porphyria*	Azo and anthraquinone dye decolorization under oxidation condition	4 heme-peroxidases, 33 CAZymes, 2 laccases, 19 H_2_O_2_-producing enz-ymes, 4 benzoquinone oxidoreductases	[6]

The electron-withdrawing nature of the azo dyes makes them unpreferred substrates for utilization as a sole source of carbon, nitrogen, or energy by microbes. Thus, the augmentation of a co-substrate becomes a requisite in biological dye degradation. However, this can further lead to an increase in the cost of technology when applied on a large scale. In this regard, a bacterial consortium (designated as SCP) was developed, which was proficient in the dye degradation in the presence of an inexpensive co-substrate (i.e., glycerol). The consortium consisting of three bacterial members and designated as ‘SCP’ based on the first letter of the genus name of each member (i.e., *Stenotrophomonas acidaminiphila* APG1, *Pseudomonas stutzeri* APG2 and *Cellulomonas* sp. APG4) was developed from a mixed-culture enriched on medium containing 100 mg Reactive Blue 28/L and 0.1% (v/v) glycerol as a co-substrate under the anoxic condition with repeated transfers for several months. The three isolates from the mixed culture were selected based on the functional and social-behavioral traits [24]. In order to gain a deeper understanding about the role of each member of the consortium in dye degradation, the genomes of all the three members of consortium SCP were sequenced and analyzed. This study aims to propose the role of different members of the bacterial consortium SCP for the degradation of azo dye utilizing glycerol as a co-substrate.

## Results

### Mediated versus non-mediated decolorization of RB28 by consortium SCP

**Fig. 1:**
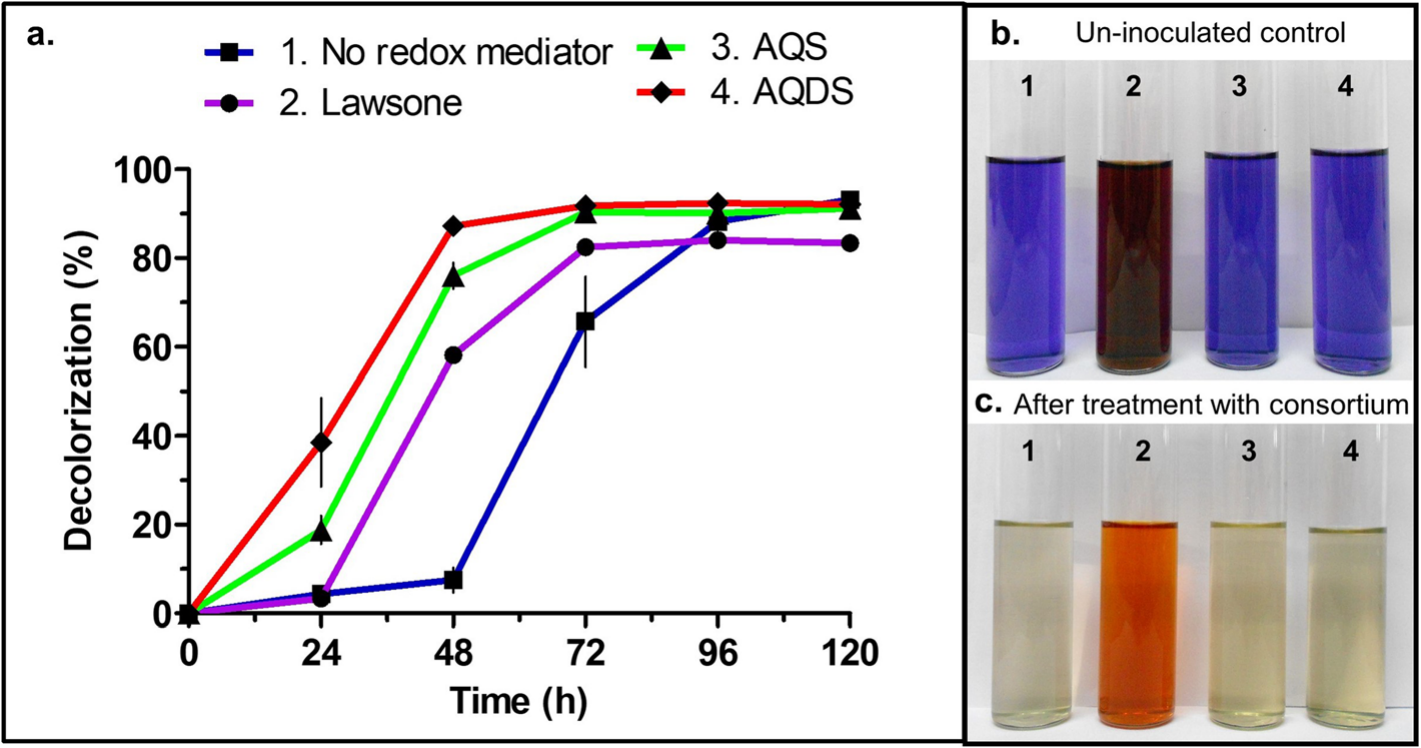
Influence of redox mediators on decolorization profile of RB28 (100 mg L-1) by SCP consortium (**a**). The color plate shows the difference in the color of dye supplemented media amended with various redox mediators; **b** untreated and **c** treated with the consortium SCP

The consortium SCP was able to decolorize up to an average of 92.08% of 100 mg RB28 L^− 1^ in 96 h under static conditions in the absence of any extraneous redox mediators. A lag phase in decolorization of up to 48 h of incubation was observed, which may be attributed to the delay in growth of a member of the consortium responsible for causing reduction of the chromophore group of the dye and thus the decolorization of the medium. Alternately, it may be due to a delay in expression of decolorization-associated enzymes or probably due to the time required to create a reductive environment favorable for the degradation of the azo bond. This initial lag was followed by exponential decolorization resulting in complete decolorization of RB28 dye during the next 48 h (Fig. 1). The augmentation of redox mediators that shuttle electrons from donors to acceptors has been shown to enhance the rate of dye decolorization [25]. Thus, in the current study, the rate of chromophore reduction (i.e., here electrophilic azo bond) was ameliorated by supplementing artificial quinone-based redox mediators viz., AQDS, AQS, and lawsone to the decolorization system. The AQDS was found to improve the initial rate (*R*_*0*_) of dye decolorization by 3.56-fold (i.e., 0.0185 mg RB28.mg biomass^− 1^.h^− 1^) in comparison to the rate of non-mediated reduction (i.e., 0.0052 mg RB28.mg biomass^− 1^.h^− 1^). Moreover, AQDS proved to be the best in accelerating dye-reduction, followed by AQS (0.0161 mg RB28.mg biomass^− 1^.h^− 1^) and lawsone (0.0087 mg RB28.mg biomass^− 1^.h^− 1^). Interestingly, no lag phase in decolorization was observed in the presence of AQDS and AQS, whereas a lower lag of up to 24 h in decolorization was found in the presence of lawsone. The UV-visible spectra of the cell-free supernatant obtained before and after RB28 decolorization exhibited the decrease in intensity of the dye chromophore band accompanied by the appearance of absorbance bands at lower wavelengths upon dye decolorization (Additional file 1). For a molecule to act as an ideal redox mediator in dye reduction, it should possess standard redox potential (${E}_0^{\prime }$) higher than that of the azo dye to be reduced and not much lower than − 320 V, i.e., ${E}_0^{\prime }$ of NAD(P) H [9]. In the present study, the following trend in the efficiency of redox mediators was observed (values between brackets indicate *R*_*0*_ in mg RB28/mg biomass/ h): AQDS (0.0185), AQS (0.0161) > Lawsone (0.0087). The trend of reduction rate was not well-corroborated with the trend of the ${E}_0^{\prime }$ of the redox mediators (values between bracket indicate ${E}_0^{\prime }$ in mV): Lawsone (− 139) > AQDS (− 184) > AQS (− 218). Van der Zee and Cervantes (2009) reported AQS to be a more strong redox mediator than AQDS due to the absence of an additional sulphonate group, which makes it more accessible, unlike AQDS [9]. However, during mediated decolorization by consortium SCP, the AQDS exhibited higher efficiency of electron shuttling, suggesting that mediated decolorization efficiency seems to be influenced not only by the standard redox potential of mediator alone but also depend on the accessibility of mediator by the cell and/or by standard redox potential of the electron donor.

### Genome features and phylogenetic analysis

The genome sequencing of *Stenotrophomonas acidaminiphila* APG1, *Pseudomonas stutzeri* APG2 and *Cellulomonas* sp. APG4 using Illumina Miseq platform produced a total of 2,896,177 paired-end reads leading to an average genomic coverage of 230.69 X, 239.46 X and 239.35 X, respectively. The assembly details and key features of the genome sequences of APG isolates are summarized in Table 2. In order to link to the biological function or structure, the genes from the three draft genomes were categorized into 25 subsystems using the RAST server [26]. The highest number of CDS were assigned to the subsystem “amino acids and derivatives” for APG1 and APG2 and “carbohydrates” for APG4 (Additional file 2). These set of metabolic genes could be critical in providing energy essential for the degradation process. Since the azo bond reduction depends on the electron donation by the degraders, the features assigned to “electron-donating reactions” in subsystem “respiration” were analyzed. It was found that APG4 had maximum features (i.e., 33) for electron donation, followed by APG1 (i.e., 29) and APG2 (i.e., 19). On gaining electrons, the dye gets primarily reduced to the corresponding aromatic amines. The subsequent transformation of the resulting amino group is subject to the nitrogen metabolizing capabilities of the organism. Thus, analysis of the features associated with “metabolism of aromatic compounds*”* and “nitrogen metabolism” was performed, and it was revealed that APG2 was highly proficient in the same as APG1 and APG4. Moreover, exposure to azo dyes can lead to oxidative stress in the microbial cells [27]. APG1 and APG2 had a very high number of features assigned to “oxidative stress”, i.e., 42 and 43, respectively, whereas APG4 had only eight features assigned to it.

**Table 2:** Genome statistics and features of *Stenotrophomonas acidaminiphila* APG1, *Pseudomonas stutzeri* APG2 and Cellulomonas sp. APG4

Attribute	***Stenotrophomonas acidaminiphila*** APG1		***Pseudomonas stutzeri*** APG2		***Cellulomonas*** sp. APG4	
	Value	% of Total	Value	% of Total	Value	% of Total
Genome size	4,102,834 bp	100	4,699,510 bp	100	3,743,932 bp	100
G + C content	2,830,955 bp	69.0	2,974,790 bp	63.30	2,751,790 bp	73.5
No. of contigs	96	NA	61	NA	48	NA
No. of subsystems	301	NA	372	NA	270	NA
Largest contig	222,264 bp	NA	276,632 bp	NA	452,363 bp	NA
N50	91,288 bp	NA	198,959 bp	NA	136,916 bp	NA
L50	15	NA	10	NA	9	NA
Secondary metabolites	78,750 bp	1.92	132,249 bp	2.81	58,048 bp	1.55
Genome islands	553,777 bp	13.50	616,150 bp	13.11	412,247 bp	11.01
Total genes	4008	100.00	4682	100.00	3704	100.00
Protein-coding sequences	3857	96.23	4537	96.90	3622	97.79
Number of GO annotations	5930	NA	7069	NA	4496	NA
tRNAs	57	1.42	54	1.15	48	1.30
rRNAs	5	0.12	6	0.13	3	0.08
ncRNA	4	0.10	4	0.09	3	0.08
Pseudogenes	53	1.32	101	2.16	55	1.48
Tandem repeats	314	7.83	102	2.18	540	14.58
CRISPR	4	0.10	3	0.06	2	0.05
CAS	0	0.00	0	0.00	0	0.00

**Fig. 2:**
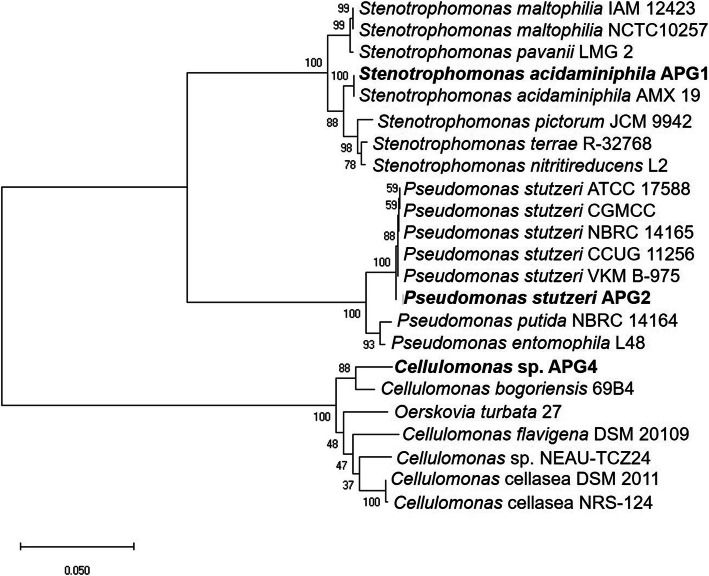
Phylogenetic analysis of APG isolates based on the 16S rRNA gene sequence in MEGA X. Tamura-Nei method was used to calculate the evolutionary distances at the bootstrap of 3000 which is indicated at the branch points. The scale bar specifies that 0.05 substitutions were present per site of nucleotide

**Fig. 3:**
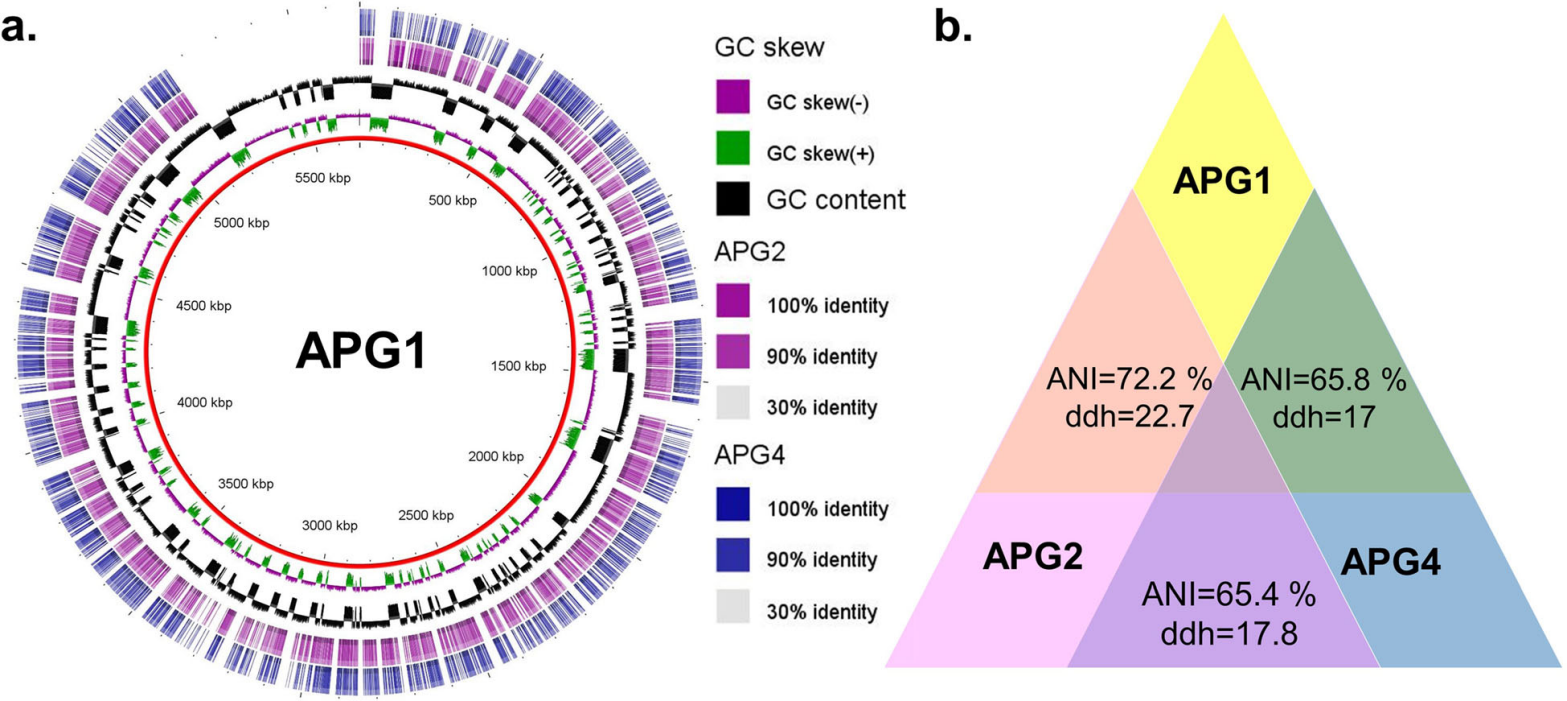
Comparison of draft genome sequences of APG isolates. **a** Schematic depiction of genomes of AGP isolates. The rings from outside to inside represent the hits recognized through blastx comparisons of *Cellulomonas* sp. APG4 (Circle 1) and *Pseudomonas stutzeri* APG2 (Circle 2) against *Stenotrophomonas acidaminiphila* APG1. Circle 3 represents GC skew (**b**) The average nucleotide identity (ANI) and in silico DNA-DNA hybridization (DDH) values amongst genomes of APG isolates

Based on the relatedness of the 16S rRNA gene sequence, the phylogenetic reconstruction of the three APG isolates with the closest type strains was conducted (Fig. 2). The APG isolates were separated into three different clades at high bootstrap support (bootstrap = 100 for each clade). The isolate APG1 was closely related to several strains of *Stenotrophomonas acidaminiphila* AMX19*.* The isolate APG2 was closely related to various strains of *Pseudomonas stutzeri,* whereas the isolate APG4 was closely related to *Cellulomonas bogoriensis* 69B4. The evaluation of phylogeny revealed that APG1 and APG2 were more closely related as they belonged to sister clades, whereas the clade of APG4 was relatively distant from these two. The circular map depicting genome comparisons was generated by BLAST Ring Image Generator (BRIG) using the APG1 draft genome as reference (Fig. 3a). The genome relatedness as established by digital DNA: DNA hybridization (dDDH) and average nucleotide identities (ANI) revealed that APG1 and APG2 shared a higher degree of similarity (dDDH = 22.7, ANI = 72%) (Fig. 3b). In contrast to this, the dDDH and ANI values of APG4 vs. APG1 (dDDH = 17, ANI = 65.7%) and APG4 vs. APG2 (dDDH = 17.8, ANI = 65.4%) were relatively lower, which correlated to 16S rRNA based phylogeny.

### Orthologous genes

The protein-coding sequences (CDS) of all three isolates were assigned to 20 different COG (cluster of orthologous groups) categories involved in information storage and processing, cellular processing and signaling, and metabolism. A total of 2975, 3695 and 2810 CDS were assigned from the draft genome of APG1, APG2 and APG4, respectively. The details of the functional classification of coding sequences to COG categories are provided in Table 3. The most significant proportion from all three genomes was dedicated to *metabolism,* i.e., 28.85% in APG1, 33.2% in APG2, and 33.62% in APG4. In APG1 and APG2, many coding sequences were assigned to category M (cell wall/membrane/envelope biogenesis, i.e., 237) and C (energy production and conversion, i.e., 260), respectively. In APG4, maximum CDS were associated with category K (transcription, i.e., 282). The genes responsible for the functions like “intracellular trafficking, secretion, and vesicular transport (U)”, and “secondary metabolites biosynthesis, transport and catabolism (Q)” might be related to dye degradation [28]. The maximum number of genes assigned to these functions were identified in APG2, followed by APG1 and APG4. This further highlights the potential of APG2 in material transport and catabolic reactions related to dye degradation.

**Table 3:** Functional classification of genes to COG categories from the draft genome of APG isolates

COG Categories	Gene number assigned to isolates		
	APG1	APG2	APG4
**Information Storage and Processing**			
Transcription (K)	200	245	282
Translation, ribosomal structure and biogenesis (J)	186	194	160
Replication, recombination and repair (L)	182	222	187
RNA processing and modification (A)	1	1	0
Total	569 (17.5%)	662 (16.5%)	629 (20.8%)
**Cellular Processes and Signaling**			
Cell wall/membrane/envelope biogenesis (M)	237	219	143
Signal transduction mechanisms (T)	134	188	144
Posttranslational modification, protein turnover, chaperones (O)	113	131	101
Defense mechanisms (V)	97	43	83
Intracellular trafficking, secretion, and vesicular transport (U)	57	101	27
Cell cycle control, cell division, chromosome partitioning (D)	46	54	38
Cell motility (N)	40	46	27
Total	724 (22.3%)	782 (19.5%)	563 (18.6%)
**Metabolism**			
Amino acid transport and metabolism (E)	207	255	193
Inorganic ion transport and metabolism (P)	174	247	155
Energy production and conversion (C)	146	260	171
Coenzyme transport and metabolism (H)	102	144	89
Carbohydrate transport and metabolism (G)	97	130	235
Lipid transport and metabolism (I)	90	120	71
Nucleotide transport and metabolism (F)	75	104	71
Secondary metabolites biosynthesis, transport & catabolism (Q)	46	72	33
Total	937 (28.9%)	1332 (33.2%)	1018 (33.6%)
**Poorly Characterized**			
Function unknown (S)	745	919	600
Total	745 (22.9%)	919 (22.9%)	600 (19.8%)
**Total no. of genes**	**2975**	**3695**	**2810**

### Antibiotic resistance determinants and secondary metabolites

The analysis of genomes by the Comprehensive Antibiotic Resistance Database (CARD) [29] revealed the presence of a few antibiotic resistance genes in the isolate APG1 and APG2. Three genes were identified in the APG1 genome, which possessed 100, 80.98 and 42.95% identity with *catB3, sul1* and *adeF* genes, respectively. The *catB3* encodes chloramphenicol acetyltransferase (CAT), whereas *sul1* encodes sulfonamide-resistant dihydropteroate synthase conferring protection against phenolic and sulfonamide antibiotics, respectively. The *adeF* encodes for a resistance-nodulation-cell division (RND) antibiotic efflux pump, which imparts resistance against fluoroquinolone and tetracycline. In APG2, two antibiotic resistance genes were predicted, and both of them were identical to the *adeF* gene, with 66.13 and 41.57% similarity. The presence of these genes indicated that the isolate might be resistant to fluoroquinolone and tetracycline. In contrast to gram-negative isolates APG1 and APG2, no antibiotic resistance genes could be predicted in gram-positive isolate APG4 (Table 4). Several diverse secondary metabolite pathways were detected from the genomes through antiSMASH analysis. APG1 genome possessed gene clusters for the production of hserlactone, lassopeptide and arylpolyene, i.e., xanthomonadin. The genome of APG2 had characteristic biosynthetic clusters for bacteriocin production, siderophore desferrioxamine, N-acetylglutaminylglutamine amide (NAGGN), carotenoid, NRPS-like ectoine, and a beta-lactone. Moreover, the gene clusters for the synthesis of a type-III polyketide synthase, alkylresorcinol and a carotenoid were found in the draft genome of APG4. A total of 2.8% genome of APG2, 1.9% genome of APG2 and 1.5% genome of APG4 corresponds to these gene clusters (Table 1).

**Table 4:** Antibiotic resistance and secondary metabolites encoded by draft genome sequences of APG1, APG2 and APG4

Traits	***Stenotrophomonas acidaminiphlia*** APG1	***Pseudomonas stutzeri*** APG2	***Cellulomonas*** sp. APG4
**Antibiotic resistance**	Resistant against phenicol, sulfonamide, sulfone, fluoroquinolone, tetracycline	Resistant against fluoroquinolone, tetracycline	None
**Secondary metabolites**	Xanthomonadin (Arylpolyene)	Desferrioxamine (Siderophore)	Alkylresorcinol (T3PKS)
	Lassopeptide	Bacteriocin	Carotenoid
	Hserlactone	N-acetylglutaminylglutamine amide	
		Ectoine (NRPS-like)	
		Carotenoid (Terpene)	
		Betalactone	

*Key*: *T3PKS* Type III polyketide synthase, *NRPS-like* Non-ribosomal peptide synthetase cluster like fragment

### Mobile genetic elements

With the help of CRISPRCas finder [30], 4, 3 and 2 putative CRISPR sequences were identified in the genome of APG1, APG2 and APG4, respectively (Table 2). However, no sequence encoding for the Cas protein could be identified in any of the genomes. The genomic islands, which are indicative of genes procured by horizontal gene transfer (HGT), were investigated in all the isolates by IslandViewer 4 [31, 32]. The abundance of genomic islands in APG1, APG2 and APG4 were traced by reordering contigs against the reference genomes *Stenotrophomonas acidaminiphila* SUNEO, *Pseudomonas stutzeri* A1501 and *Cellulomonas flavigena* DSM 20109, respectively (Additional files 3 and 4). APG1 and APG2 consist of 38 islands each, whereas the APG4 draft genome consists of 21 genomic islands (Table 4). A highly diverse set of genomic islands were detected in the genomes ranging from 4163 bp to 110,649 bp in size (Additional file 5)**.** In APG1, 13.5% of the genome is represented by dispersed genomic islands, encoding a total of 506 different proteins. The CDS for several transcriptional regulators, membrane proteins (e.g. copper homeostasis membrane protein CopD), and other proteins, including diacylglycerol kinase, nitrogen fixation protein FixH and sulfite exporter TauE/SafE family proteins were identified. Amongst all the isolates, APG2 encoded maximum number of proteins in its genomic islands (i.e., 591), accounting for 13.11% of the genome. The CDS for cyanate hydratase (EC 4.2.1.104), aerotaxis sensor receptor protein, glycerate kinase (EC 2.7.1.31), copper-sensing protein, copper-translocating ATPase (EC 3.6.3.3), and several other transporters were detected in the genome islands of the APG2 genome. Approximately 11.01% of the APG4 genome was dedicated to the islands, encoding up to a total of 378 proteins. The proteins encoded by these islands included carbohydrate ABC transporter permease and substrate-binding protein, two-component sensor histidine kinase, chemotaxis protein CheA/CheW, and heavy metal translocating P-type ATPase, etc. Interestingly, three organisms consisted of a completely distinct array of proteins in their genomic islands except for a DEAD/DEAH box helicase and a copper resistance system multi-copper oxidase. DEAD/DEAH box helicases play a crucial role in the post-transcriptional regulation, whereas copper resistance system multicopper oxidase is involved in copper homeostasis in bacteria [33, 34]. Since the azo dye taken for the study was copper-complexed, it seems more likely that the resistance to the heavy metal might have contributed to selection of these organisms during the enrichment of a mixed culture. Overall, the distinctive set of genomic islands indicate the absence of active horizontal transfer activities between the isolates of the consortium.

A large number of insertion sequences (ISs) were also identified from the genomes of all three organisms. APG1 draft genome consisted of 28 ISs: ten belonging to the Tn3 family and seven to the ISL3 family (Additional file 6). APG2 genome harbored 50 ISs, including 16, 15 and 9 sequences from IS3, IS5 and ISL3 family, respectively. The lowest number of sequences were detected in the APG4 genome, i.e., 21, the majority belonging to the Tn3 family (i.e., 4). On comparing ISs from the three bacterial species, it was apparent that sequences of Tn3, IS3 and ISL3 families were commonly prevalent in all. However, each isolate had a unique set of sequences. Different regions of two ISs, i.e., ISAz517 and ISArsp9, were found in APG1 and APG4 draft genomes. Also, distinct portions of ISPsy43 were identified in the genome of APG2 and APG4. At least one large intact prophage (PHASTER score > 90) of 19.9 Kb encoding for terminases, phage tail, plate, fiber, lysis, and coat proteins, was traced in the APG2 genome (Table 5). It also encoded several hypothetical and phage-like proteins. The genome of APG1 possessed one incomplete prophage (PHASTER score < 70) of 5.7 Kb, whereas APG4 had two incomplete prophages of 6.1 Kb each. These were categorized under incomplete status as their intactness was dubious. APG1 prophage encoded for several hypothetical and phage-like proteins. One of the prophage sequences from APG4 consisted of CDSs of terminases, phage portal, coat, and hypothetical proteins, whereas the other prophage sequence seems to encode several hypothetical and phage-like proteins.

**Table 5:** Details of phage genes and genomic islands identified in the APG genomes by PHASTER and IslandViewer 4, respectively

Genes	APG1	APG2	APG4
**Phage**			
Intact (score > 90)	–	1	–
Questionable (score 70-90)	–	–	–
Incomplete (score < 70)	1	–	2
Total Size (Kb)	5.7	19.9	12.2
**Genomic islands**			
Number	38	38	21
Total Size (Kb)	25.8	40.7	28.4

### Carbohydrate active enzymes

**Fig. 4:**
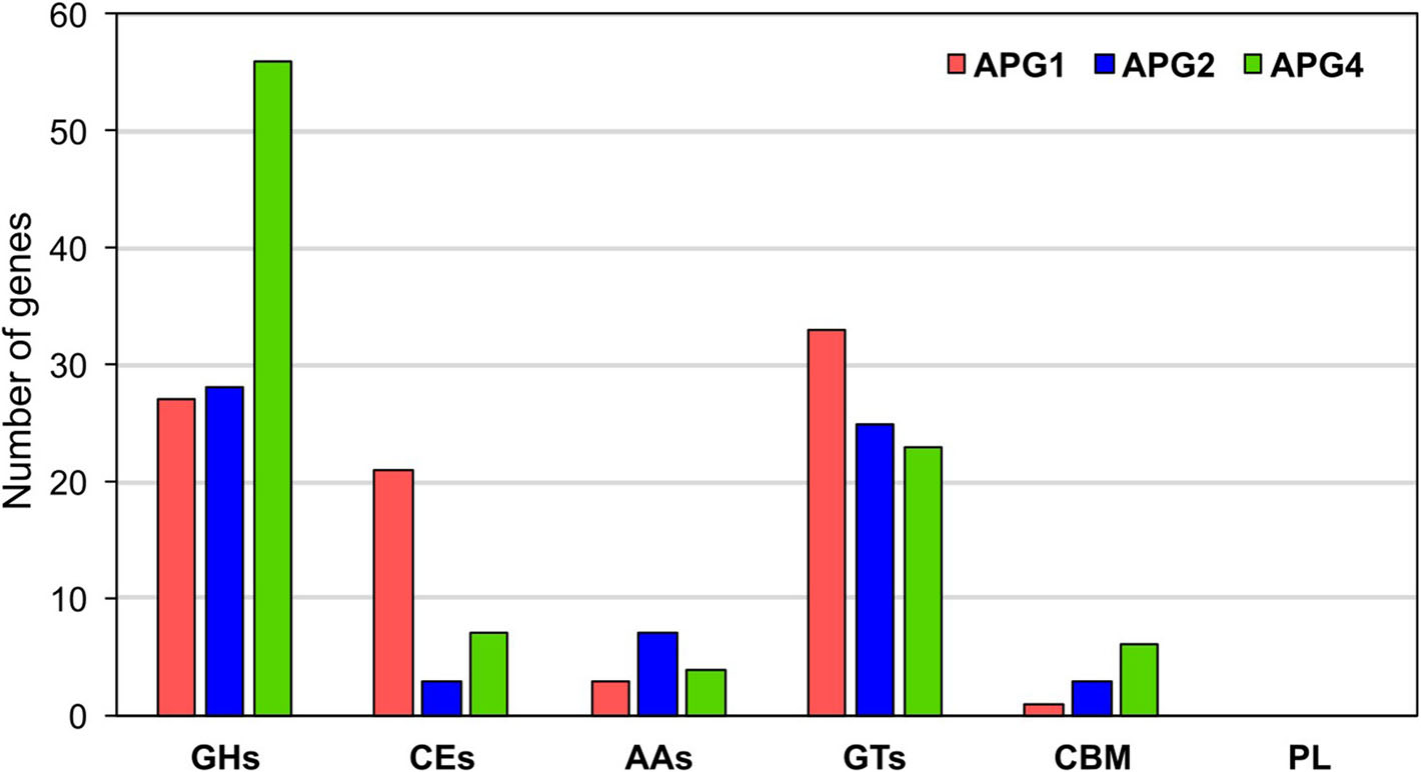
Distribution of CAZymes across the three isolates. GH, Glycoside hydrolases; CE, Carbohydrate esterases; AA, Auxiliary activities; GT, Glycosyl transferases; CBM, Carbohydrate-binding molecule; PL, Polysaccharide lyases

The investigation of CAZymes from the three genomes revealed high diversity in the enzymes related to the anabolism of carbohydrates. APG4 consisted of the highest number of CAZymes, i.e., 96, followed by APG1 and APG2 with 85 and 66 enzymes, respectively. Of all the categories, APG4 was most abundant in glycoside hydrolases (i.e., GHs = 56), followed by glycosyl transferases (i.e., GTs = 23) as represented in Fig. 4. Also, in APG4, the glycoside hydrolases from 30 different families were identified, which further suggests the isolate’s ability to utilize a wide variety of polysaccharides. The maximum number of carbohydrate-binding domains (CBDs) (i.e., 6) was identified in APG4. The APG1 possessed the highest number of glycosyl transferases (i.e., GTs = 33) and carbohydrate esterases (i.e., CEs = 21). Interestingly, as compared to the other two isolates, APG2 had the highest number of CAZymes with auxiliary activities (i.e., AA = 7). The proteins categorized in the auxiliary activities category are the redox enzymes that function concomitantly with CAZymes, specifically in lignin breakdown [35]. These ligninolytic enzymes might also be contributing to dye degradation. Besides, no enzyme was detected in the polysaccharide lyase (PL) category from any of the genomes. The analysis of these enzymes revealed the carbohydrate degrading potential of the three organisms.

### Integrated model for glycerol utilization

**Fig. 5:**
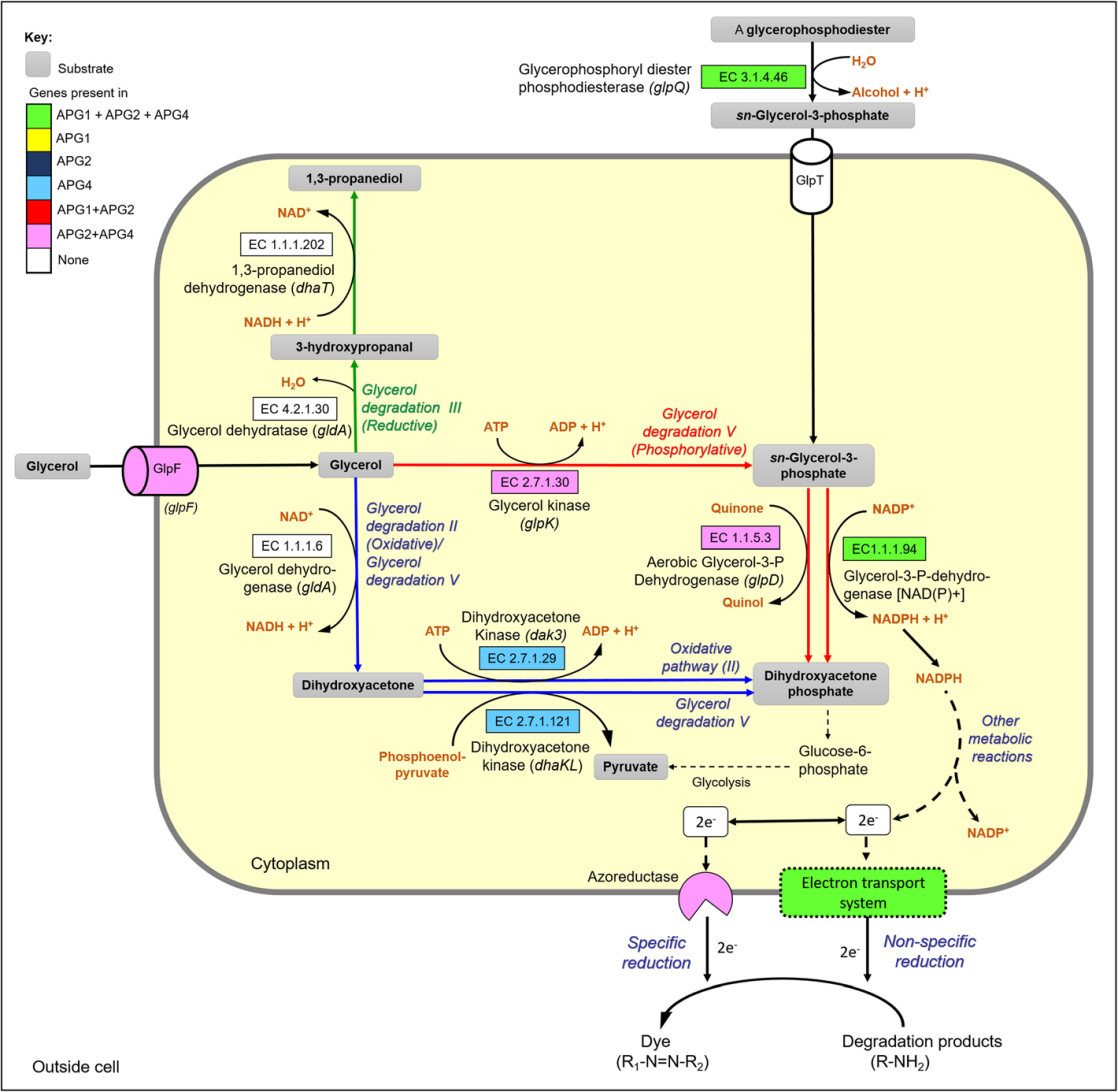
Overview of glycerol uptake and metabolism in the three organisms. The color in the background represents the combinations of genomes possessing the coding sequences for that respective enzyme. Solid arrows represent one-step reaction whereas dashed-arrow represents multiple steps of the pathway

The consortium SCP was able to reduce the azo dye (RB28) in the presence of an inexpensive co-substrate, i.e., glycerol. Therefore, the glycerol utilization ability of all the members of the consortium was analyzed. The isolates were cultured on minimal media containing glycerol as a sole source of carbon and energy, and it was observed that *Pseudomonas stutzeri* APG2 exhibited good growth, followed by *Cellulomonas* sp. APG4. Although *Stenotrophomonas acidaminiphila* APG1 was unable to grow on glycerol-containing plates, it exhibited growth when co-inoculated in the vicinity of the other two isolates in the cross-feeding assay; suggesting its dependence of the other members of the consortium for growth (Additional file 7). Also, the higher growth of APG4 was observed in the proximity of APG2 on the glycerol agar plate. Therefore, the draft genomes of all isolates were assessed to trace glycerol’s fate during the decolorization by SCP. The glycerol concentrations across the membrane are equilibrated by a glycerol uptake transmembrane protein, GlpF, which facilitates the glycerol transport inside the cell (Fig. 5) [36]. Immediately upon entering the cell, the glycerol is catabolized by different pathways depending on the organism’s genetic composition. One of the most common aerobic pathways (glycerol degradation I) involves phosphorylation of glycerol to *sn*-glycerol-3-phosphate (G3P) by an ATP-dependent enzyme glycerol kinase (EC 2.7.1.30) encoded by *glpK* gene [37, 38]. The facilitator protein cannot diffuse glycerol-3-phosphate (G3P) outside the cell, and thus, G3P resides in the cell, followed by its oxidation to dihydroxyacetone phosphate (DHAP) by a homo-dimeric enzyme, glycerol-3-phosphate dehydrogenase (EC 1.1.5.3) encoded by *glpD* gene [37]. This phosphorylative pathway dependent on the *glp* system is reported in several organisms, including *Escherichia coli, Cellulomonas* sp. NT3060, etc. [39]. In *E. coli,* the expression of the *glp* regulon is under the control of an inducer, G3P, and a repressor, GlpR [40]. The isolates APG2 and APG4 consist of genes encoding glycerol uptake facilitator protein (*glpF*), glycerol kinase (*glpK*) (EC 2.7.1.30), glyceraldehyde-3-phosphate dehydrogenase (*glpD*) (EC 1.1.5.3), and glycerol-3-phosphate regulon repressor (*glpR*); thus indicating the efficient uptake and dissimilation of glycerol by phosphorylative pathway. In contrast to this, APG1 lacked these three genes indicating its deficiency in importing and metabolizing glycerol by this pathway.

Another pathway, i.e., glycerol degradation II includes oxidation of glycerol to dihydroxyacetone (DHA) by NAD^+^-dependent glycerol dehydrogenase (EC 1.1.1.6; encoded by *gldA* gene) under anaerobic condition. DHA is further converted to DHAP by ATP-dependent DHA kinase (EC 2.7.1.29; encoded by *dhaK*) [38, 39]. Moreover, this oxidative pathway under anaerobic conditions is coupled to a reductive pathway, which was first discovered in *Klebsiella pneumonia* ATCC 8724 [41]. This reductive process, i.e., glycerol degradation III, is a dismutation reaction involving glycerol dehydration to 3-hydroxypropanal (3-HPA) by glycerol dehydratase (EC 4.2.1.30). 3-HPA is further fermented to 1,3-propanediol (PDO) by an NADH-associated 1,3-propanediol dehydrogenase (EC 1.1.1.202) [42]. The four enzymes involved in both pathways, i.e., glycerol dehydrogenase (EC 1.1.1.6), DHA kinase (EC 2.7.1.29), glycerol dehydratase (EC 4.2.1.30), and 1,3-propanediol dehydrogenase (EC 1.1.1.202) are encoded by *dha* regulon [43]. The expression of these enzymes is induced in the presence of DHA or glycerol [44]. Also, the enzymes glycerol dehydratase and 1,3-propanediol dehydrogenase are used for the commercial production of PDO [45]. Other organisms possessing *dha* regulon include *Clostridium butyricum, Citrobacter freundii*, *Clostridium pasteurianum*, etc. [41]. APG1 and APG2 were deficient in all four enzymes, implying the absence of glycerol utilization by this pathway, whereas APG4 consisted of a few CDS for ATP-dependent DHA kinase (EC 2.7.1.29) (Fig. 5).

An additional dehydrogenation pathway for glycerol utilization (i.e., glycerol degradation V) involves the conversion of glycerol to DHA by glycerol dehydrogenase, followed by phosphorylation of DHA to DHAP by a PEP-dependent DHA kinase (EC 2.7.1.121; encoded by *dhaKL*) [42, 43]. The genes for PEP-dependent DHA kinase (EC 2.7.1.121) were exclusively identified in the APG4 genome. Except for the reductive pathway, conversion of glycerol to DHAP further leads to its metabolism through the glycolytic pathway. Moreover, glycerophosphoryl diester phosphodiesterase (EC 3.1.4.46) is a periplasmic enzyme responsible for the hydrolysis of glycerophosphodiester to *sn*-glycerol-3-phosphate and an alcohol [46]. This enzyme was identified in all the genomes. GlpT, a glycerol-3-phosphate transporter involved in the uptake of glycerol-3-phosphate from the periplasm into cells [47], was absent in all the genomes. All the pathways for glycerol uptake and metabolism are summarized in Fig. 5, and the total number of ORFs for enzymes concerning the same are provided in Additional file 8. Thus, the genome analyses of the APG isolates well-corroborated with the experimental results i.e., APG1 was unable grow on glycerol as sole carbon source and its dependence on the APG2 and/or APG4 for the growth was demonstrated by cross-feeding experiment.

### Dye degrading enzymes

**Fig. 6:**
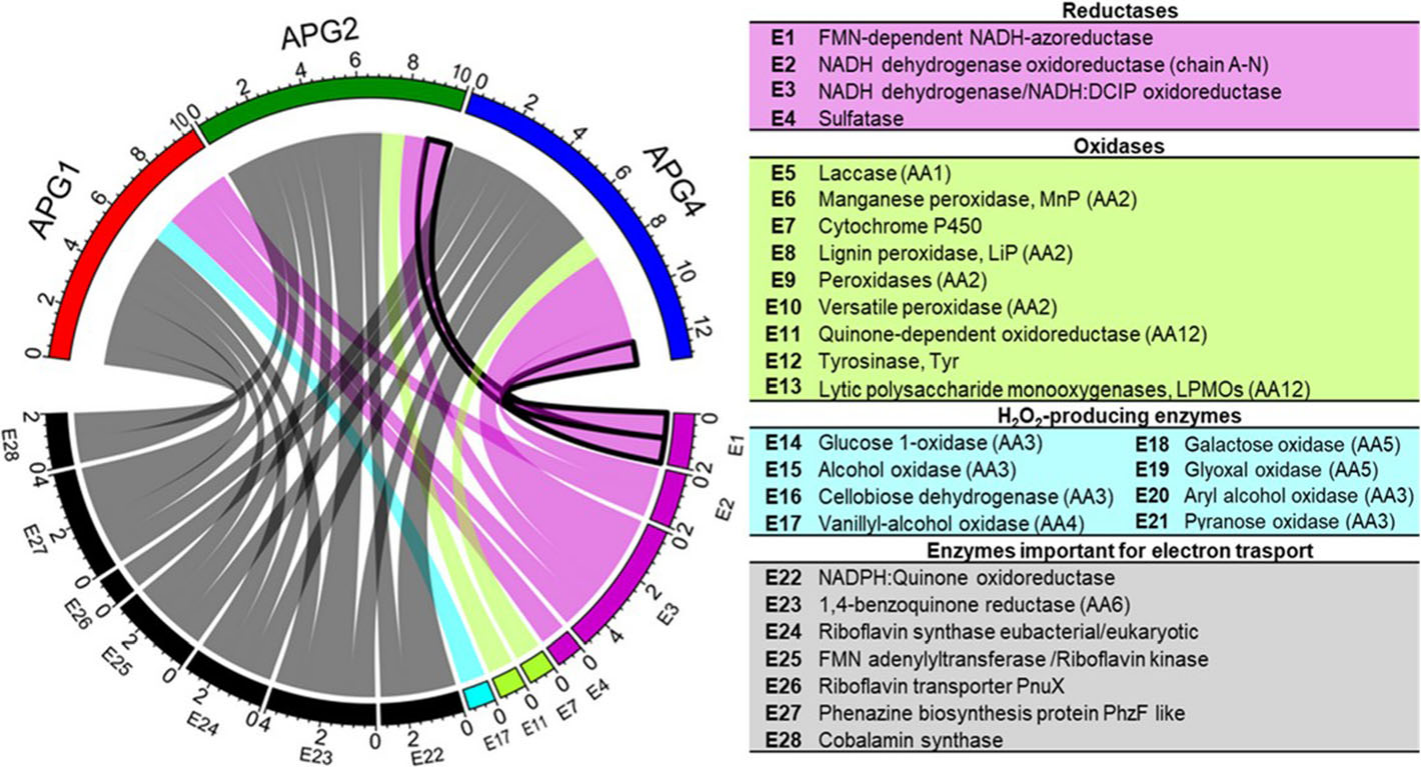
Circos plot representing the abundance of RB28 degradative enzymes in the members of consortium SCP. The sectors in the outer circle at the top indicate the draft genomes of APG isolates i.e., APG1 (red), APG2 (green) and APG4 (blue) whereas at the bottom indicate different enzymes. The width of the ribbons represent the relative abundance of the coding sequences identified in the genomes. The different groups of enzymes are color-coded and the key-enzyme involved in dye degradation i.e., FMN-dependent NADH-azoreductase has been highlighted

Under anoxic conditions, the breakdown of azo dyes occurs by gratuitous reduction of the extremely electronegative chromophore, i.e., azo bond (−N=N-), forming substituted or non-substituted aromatic amines as intermediates [48]. The reduction may take place via several means, i.e., by enzymes, redox mediators, chemical reductants arising from biogenesis (e.g., sulfide), or synergistically by all of them [49]. The colorless amines formed may then be mineralized by the subsequent oxidative reactions [50]. Thus, the APG genomes were analyzed for the presence of enzymes and redox intermediaries that are actively involved in the breakdown of the azo bridge of dye. Since the color removal by consortium SCP occurred under anoxic conditions, the organisms were primarily assessed for the reductase genes. FMN-dependent NADH azoreductase (EC 1.7.1.6) was identified from the draft genomes of APG2 and APG4, suggesting their role in the dye decolorization process (Fig. 6). Previously, it has been reported that APG4 possessed the NADH-dependent MR reductase activity [24]. Two types of NADH dehydrogenases involved in the dye-reduction were detected in the members of the consortium. The NADH dehydrogenase oxidoreductase (EC 1.6.5.3) was detected in the APG1 and APG4 genome, whereas another NADH dehydrogenase, also known as NADH: DCIP oxidoreductase (EC 1.6.99.3), was identified in all three organisms. APG4 contained the highest number of ORFs for NADH: DCIP oxidoreductase (i.e., 3), thus indicating its greater contribution in the degradation of dye under the reductive condition as compared to the others. These results corroborated with previously reported NADH-DCIP reductase activities of APG isolates [24]. Amongst all isolates, APG4 possessed more than 18-fold higher reductase activity in comparison to APG1 and APG2. The sulfatase enzyme that catalyzes the hydrolysis of sulfate esters in complex molecules was identified only in the genome of APG1 (Additional file 9), suggesting its role in the desulfonation of vinyl sulphone moiety present in RB28. Under aerobic conditions, the enzymes viz., laccase (Lac), manganese peroxidase (MnP), tyrosinase (Tyr), lignin peroxidase (LiP), hexane oxidase and cellobiose dehydrogenase are implicated in catalyzing oxidative dye degradation [5]. Since the RB28 decolorization by consortium SCP occurred under anoxic conditions, the oxidases might not be responsible for dye decolorization. However, these might be involved in the partial mineralization of aromatic amines. Thus, the genomes of APG isolates were examined for the presence of oxidases. Laccases are copper-containing oxidases catalyzing the oxidation of aromatics coupled to the synthesis of water as a by-product [51]. Notably, none of the genomes possessed genes encoding this enzyme which is in agreement with our experimental results [24]. However, Cytochrome P450 (CYP450) has been reported to metabolize a range of xenobiotic compounds, including dye, by hydroxylation reactions [52]. Interestingly CDS for CYP450 was exclusively found in the APG4 draft genome. The proteins identified with the auxiliary activities from the CAZyme database may involve the degradation of aromatic amines by the ligninolytic mechanism [6]. A very few proteins dedicated to this category of CAZymes were identified in the three genomes. The enzymes involved in the lignin degradation, i.e., manganese peroxidase (1.11.1.13) and lytic polysaccharide monooxygenases (LPMOs), were absent in all. However, an accessory enzyme involved in ligninolysis, i.e., quinone-dependent oxidoreductase, was found in the APG2 genome. The H_2_O_2_ producing enzymes viz., glucose-1-oxidase (EC 1.1.3.4), alcohol oxidase (EC 1.1.3.13), cellobiose dehydrogenase (EC 1.1.99.18), aryl alcohol oxidase (EC 1.1.3.7), pyranose oxidase (EC 1.1.3.10), galactose oxidase (EC 1.1.3.9), and glyoxal oxidase (EC 1.2.3.15) were absent in all. However, one CDS for vanillyl-alcohol oxidase (EC 1.1.3.38) was detected in the genome of APG1.

During dye reduction under anaerobic/anoxic conditions, the transfer of electrons is the rate-limiting step [53]. Several redox-active biological molecules, e.g., quinones, flavins, cytochromes, pyridines, porphyrins, cobalamins, phenazines, quinines, etc., can non-enzymatically improve the rate of reduction by ferrying the electrons [54]. In fungi and bacteria, the quinone and benzoquinone reductases are reported to degrade several substituted aromatics as well as reduce the metal ions like chromate [Cr (VI)] by participating in the quinone-mediated redox cycle [35, 55, 56]. Thus, the genomes were screened for the presence of 1,4-benzoquinone reductase (EC 1.6.5.6) and NADPH: Quinone oxidoreductase (EC 1.6.5.5). It was found that all APG isolates possessed the genes for these enzymes. In addition to this, riboflavins can conduct extracellular dye or iron reduction by mediating electron transfer [1, 57]. Thus, riboflavin synthase (EC 2.5.1.9), which catalyzes the final step of riboflavin formation in the cell, and riboflavin kinase (EC 2.7.1.26), which utilizes riboflavin to synthesize the coenzymes, i.e., flavin mononucleotide (FMN) and flavin adenine dinucleotide (FAD) [58], were traced in the genomes. All the genomes harbored the CDSs for these two enzymes. Moreover, APG4 consisted of a gene encoding a transmembrane protein responsible for riboflavin transport, i.e., riboflavin transporter PnuX. Phenazines are nitrogen-containing pigmented secondary metabolites involved in cellular redox-cycling [59]. All the APG isolates consisted of a gene for PhzF like phenazine biosynthesis protein, which is required to synthesize phenazines. Another such molecule that can shuttle electrons is cobalamin, and the enzyme required for its synthesis is cobalamin synthase. It was identified in the genome of isolate APG1 and APG2. Additionally, several components of cytochrome c oxidase were identified in all three genomes.

## Discussion

In the present study, the genomics approach was used to understand the role of each member of consortium SCP in the degradation of a vinyl sulfone based copper-containing mono-azo dye Reactive Blue 28 (RB28). The consortium was able to degrade 100 mg L^− 1^ of RB28 in 96 h under the static condition in the presence of glycerol as a co-substrate. The isolates *Stenotrophomonas acidaminiphila* APG1, *Pseudomonas stutzeri* APG2 and *Cellulomonas* sp. APG4 possessed a circular chromosome of ~ 4.1 Mb, ~ 4.7 Mb and ~ 3,7 Mb, respectively. Based on in silico DNA: DNA hybridization, it was apparent that the gram-negative isolates, i.e., APG2 and APG1, had higher genomic relatedness than that with gram-positive APG4. The RB28 degradation efficiency (i.e., in terms of percent degradation and rate) was found to be maximum when all three strains were present in the system. Interestingly, the same isolates could not degrade the dye individually, indicating the prevalence of strong cooperation within the members during the process [24]. The comparison of subsystems obtained from RAST revealed that APG4 might be central in dye reduction as it had maximum features for “electron-donating reactions”. Moreover, the distribution of genes in the subsystems signifies the metabolic diversity of the consortium. The tandem repeats, which play a vital role in gene regulation and evolution [60], were highest in APG4. The organisms developed for environmental applications should be devoid of antibiotic resistance genes (ARGs) [61]. APG1 and APG2 possessed only a few ARGs, whereas APG4 did not contain any, indicating the suitability of consortium SCP for environmental application. Moreover, in every environmental bacterium, antibiotic resistance is conferred by horizontal gene transfer [62, 63]. So, the location of ARGs in the genome was carefully examined, and it was revealed that *sul1* (encoding for sulfonamide-resistant dihydropteroate synthase) in APG1 was present in a genome island, implicating its acquisition horizontally from co-inhabitants in the environmental niche from where it was isolated. The functional diversity of an organism is highly governed by the genes acquired by horizontal gene transfer (HGT). Thus, the APG genomes were screened for various mobile genetic elements, including CRISPR-Cas genes, genomic islands, insertion sequences and prophages. APG2 contained the highest number of genomic islands and insertion sequences. All three members of consortium SCP possessed several carbohydrate-active enzymes. Despite the smaller genome size and lowest number of coding sequences, APG4 had the highest number of CAZymes. It was rich in glycoside hydrolases (GHs) and glycosyltransferases (GTs), which are also involved in cell-to-cell communication, sustenance of cell-structure and energy metabolism [64]. Therefore, it implies that the isolate APG4 is capable of utilizing a range of polysaccharides.

**Fig. 7:**
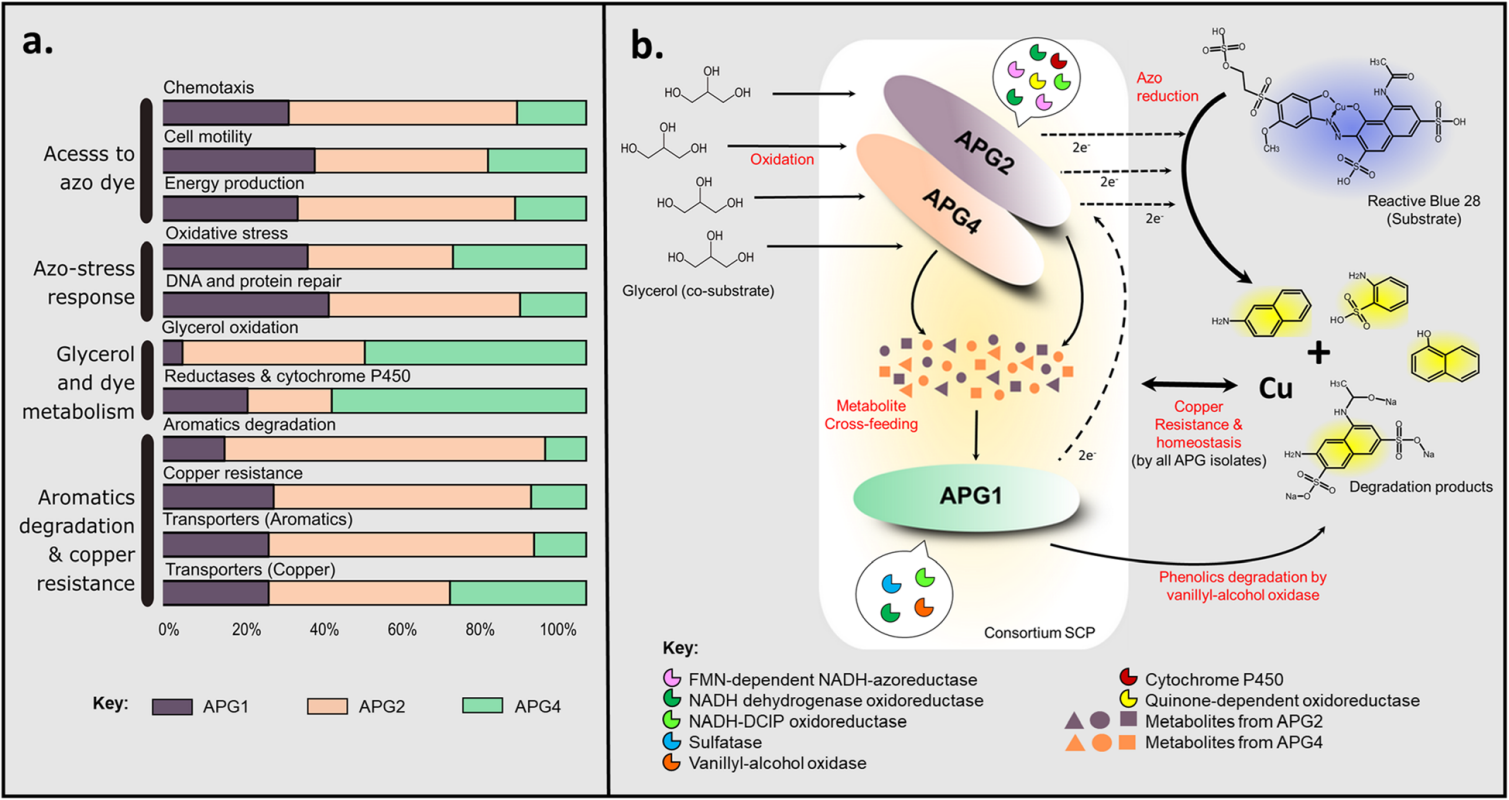
**a** Contribution of each APG isolate in RB28 degradation. **b** Model proposed to extend insights into mechanism of dye co-metabolism by the APG isolates

When the microbial cells encounter a chemical stimulus like dyes, these respond by altering physiology in multifarious ways. The previous reports on transcriptome analysis of the azo-dye respiring or degrading strains have revealed that dyes induce the expressions of genes associated with chemotaxis, signal transduction, motility, DNA and protein damage repair, and oxidative stress [65]. Based on this, a meticulous inspection of the ORFs encoding several critical proteins dedicated to these functions was conducted (Additional files 10, 11, 12, 13, 14 and 15), and a model for the mechanism of dye co-metabolism was hypothesized (Fig. 7a and b). Initially, the microbes access dye molecules, which is accompanied by an escalation in energy conservation in the cells. The conserved energy might be required for the expression of degradative enzymes and for dealing with the oxidative stress caused by the dyes. This is followed by pollutant removal with the help of degradative enzymes. Furthermore, in an attempt to achieve complete detoxification, the cells may express a set of genes required for mineralizing products/intermediates of dye reduction. Thus, a sequence of events occurs to execute the process of dye degradation and detoxification. The cellular response to dyes is primarily mediated by chemotaxis and signal transduction. The analysis of genotypic determinants of chemotaxis (Additional file 9) revealed that the APG2 had the maximum number of genes (i.e., thirty) for methyl-accepting chemotaxis protein (MCP). These proteins are elemental in beginning the cascade of chemotaxis signal transduction. On sensing the environmental signal, MCPs activate the CheA histidine kinase, which is further responsible for moderating the cellular motility towards the higher concentration of substrates. Thus, it was predicted that APG2 had either quick or greater access to dye molecule than APG1 and APG4. The other essential proteins of the cascade, i.e., CheA, CheB, CheR, CheW, CheY and CheZ, were detected in all the genomes. Also, the genes for flagellar proteins and motility regulation were most abundant in APG2 (i.e., 49), followed by APG1 (i.e., 43) and APG4 (i.e., 28). The proteins of the electron transport chain are dynamically involved in electron transfer to the extracellular substrates. Notably, the proteins of the cytochrome c family play a crucial role in energy conservation by creating a proton gradient across the membrane as well as extracellular electron transfer [66]. Thus, genes for diverse cytochromes were identified, and it was revealed that APG2 had the highest number of genes (i.e., 79) for energy conservation, followed by APG1 (i.e., 49) and APG4 (i.e., 26) (Fig. 7a; Additional file 11). Thus, it was predicted that APG2 was more efficient in energy conservation than APG1 and APG4. In the presence of pollutants like dyes, the DNA and protein get highly damaged, which triggers the damage repair mechanism in the cell [65]. Thus, the examination of chaperones, heat shock proteins, DNA ligases, polymerases, etc. was conducted. It was revealed all three genomes possessed a relatively the same number of enzymes (Fig. 7a; Additional file 12). Also, in the presence of dyes, the cells might undergo immense oxidative stress, which might induce the activities of antioxidant enzymes like catalase and superoxide dismutase [27]. When the ORFs for stress-related enzymes (including antioxidant glutathione) were compared within three genomes, it was observed that APG2 and APG1 had a higher number of such enzymes (Fig. 7a; Additional file 13).

RB28 degradation by consortium SCP was conducted in the presence of glycerol as a co-substrate. Through the co-culturing experiment, it was revealed that APG1 was unable to utilize glycerol, and thus, it was hypothesized that this member of the consortium was being cross-fed by APG2 and/or APG4 for its survival and growth. However, the exact biomolecules involved in the cooperative cross-feeding during decolorization were not identified. Also, the enhanced growth of APG4 when placed proximal to APG2 implicated the prevalence of interaction or cross-talk amongst the APG isolates atleast with respect to growth in presence of glycerol as sole carbon source. The genome mining of all the isolates was performed to reconstruct a model for glycerol metabolism. The APG2 and APG4 possess glycerol uptake protein GlpF, which was not present in APG1. In addition to the glycerol uptake genes, its catabolism by aerobic phosphorylative pathway was identified in APG2 and APG4. This is a significant finding suggesting the role of APG2 and APG4 in the abstraction of electrons from glycerol for azo bond reduction (Fig. 5). The current findings of glycerol utilization by APG4 are in agreement with a previous report where *Cellulomonas* sp. NT3060 was unable to dissimilate glycerol anaerobically but instead metabolized it under aerobic condition by *glp* system [39]. Although all three isolates were deficient in GlpT transporter, these had the potential to degrade glycerophosphodiester to *sn*-glycerol-3-phosphate by glycerophosphodiesterase (Fig. 5). This work demonstrates the coupling of glycerol oxidation to dye reduction by consortium SCP under anoxic conditions. In earlier reports, the glycerol has been exploited for simultaneous sulfate reduction by microorganisms and electricity generation in a microbial fuel cell (MFC) [67, 68]. The RB28 decolorization by consortium SCP seems to occur by reductases mediated cleavage of azo-bond under anoxic condition. The key enzyme i.e., FMN-dependent NADH-azoreductase required for the breakage of the azo bond (−N=N–) is present in both APG2 and APG4 (Fig. 6). The RB28 is a sulfonated azo dye and thus, its reduction might lead to the generation of sulfated intermediates. The enzyme, which hydrolyzes sulfate esters from complex molecules, was detected only in the genome of APG1. This may be attributed to its persistence in mixed culture during the course of enrichment on dye containing medium with glycerol as a co-substrate. Also, the molecular basis of dye and aromatic amine transformation by oxidative and H_2_O_2_-producing enzymes was evaluated. For this, the CAZymes with auxiliary activities were scrutinized and it was found that all members were deficient in most of the ligninolytic enzymes. The genes for quinone-dependent oxidoreductase and vanillyl-alcohol oxidase (AA4) were identified in the APG2 and APG1 genome, respectively. The oxidoreductase from APG2 might be involved in electron shuttling, whereas the vanillyl-alcohol oxidase from APG1 is a FAD-dependent aryl alcohol oxidase capable of oxidizing aromatic substrates with simultaneous production of H_2_O_2_ [69]. Gil et al. (2018) had reported the involvement of several enzymes with auxiliary activities in Reactive Black 5 degradation by *Trichosporon akiyoshidainum* under oxidative conditions [6]. Another vital enzyme involved in xenobiotic transformation, i.e., cytochrome P450, was identified exclusively in APG4 member of the consortium. The cytochrome P450 mediated biotransformation has been earlier reported in several organisms viz., *Bacillus megaterium*, *Rhodotorula* sp.*, Pseudomonas* sp., *Candida* sp., *Streptomyces* sp., *Shewanella putrefaciens,* etc. [70–72]. Moreover, the azo-dye reduction can be facilitated non-specifically by several redox-active molecules, and thus the use of artificial redox mediators (i.e., AQDS, AQS and lawsone) has been demonstrated to accelerate the rate of dye reduction. Microbes itself produce several molecules involved in electron shuttling and the genes responsible for the synthesis of such molecules were identified from the draft genome of each member. Also, the membrane components related to the electron transport chain can participate in the azo dye reduction [1]. The maximum number of redox-active molecules, including enzymes and membrane-bound cytochrome c oxidase, were identified in APG2 (i.e., 28). Riboflavin-related enzymes, i.e., riboflavin synthase eubacterial/eukaryotic and riboflavin kinase, were present in all the members of SCP. However, riboflavin transporter PnuX was present in APG4 only. The results emphasize the active involvement of all three isolates in the electron transfer in the community.

The reduction of azo bond under anoxic conditions engenders the formation of the chromophore-deficient secondary pollutants, i.e., aromatic intermediates mostly substituted with amines, nitro or sulfonate groups [73]. The determination of RB28 degradation pathway in consortium SCP revealed the appearance of smaller aromatics like 1-naphthylamine, aniline-2-sulphonic acid, 1-amino-8-hydroxynaphthalene 1-naphthol, etc. and some linear products like octanal [24]. These may, in turn, be more mutagenic and carcinogenic than the parent dye molecule [74]. Thus, the potential of the individual member to breakdown aromatic compounds and their transport was also examined (Additional files 14 and 15). The genes dedicated to benzoate, catechol, phenol, toluene, β-Ketoadipate (catechol branch) and protocatechuate degradation pathway were found to be present only in APG2. Interestingly, the genes for transformation of 4-aminobenzoate were identified in all the genomes whereas that of gentisate degradation were present only in APG4. The homogentisate pathway for aromatic degradation were identified in APG2 and APG1. Several specific enzymes required for the degradation of other additional twenty aromatic compounds like aniline, naphthalene, xylene, phthalate, anthranilate, benzene, biphenyl, m-cresol etc. were not detected in any isolate. Notably, all the genomes possessed genes encoding for transporters of aromatic compounds. Again, APG2 had the highest number of transporters for aromatics amongst all suggesting its putative role in oxidative catabolism of aromatic amines resulting from reductive cleavage of azo bond of azo dyes. Reactive Blue 28 is a mono-azo dye containing copper atom complexed to it. The higher concentration of copper can be toxic to the cells. Thus, genes involved in copper sensing, translocation and resistance were assessed and it was found that APG2 had maximum number of genes for regulating copper concentrations across the cell (Fig. 7a; Additional file 15). The important enzymes in this regards include copper sensing two-component system enzymes CusS and CusR, copper resistance proteins, copper homeostasis proteins and multicopper oxidase*.*

## Conclusion

This study sheds light on the mechanism of azo-dye degradation by a bacterial consortium SCP at the molecular level. The genome annotations enabled us to identify the traits of each organism and assign the role they may play in degradation of the RB28 dye, being the member of consortium (Fig. 7b). From the genome comparisons, it was apparent that *Pseudomonas stutzeri* APG2 and *Stenotrophomonas acidaminiphila* APG1 had greater ability to sense and access azo dye. Similarly, both these members were more competent in energy conservation and cellular damage repair system in comparison to *Cellulomonas* sp. APG4. All the isolates were more or less equally competent to overcome the oxidative stress. Interestingly, the key functions for the pollutant removal i.e., co-substrate oxidation and dye reduction seemed to be conducted by APG4. The isolate APG1 was incapable of directly utilizing the co-substrate, i.e., glycerol, which was also demonstrated experimentally. However, it seems to contribute electron-shuttles and play an accessory role by accelerating the decolorization of azo dye. Moreover, APG2 seems to be responsible for degradation of the aromatic amines released upon the cleavage of azo-bond, an initial step in azo dye degradation. Thus, it can be concluded that the degradation and detoxification performed by the consortium SCP is mainly due to APG2 and APG4. It is noteworthy that APG4 is abundant in genes for CAZymes, which can be exploited to extend the application of this consortium with other co-substrates. The articulation of a bacterial consortium for remediation of xenobiotic like azo dyes should not only consist of isolates possessing bountiful of degradative enzymes but also be rich in the redox-active molecules, metal transforming proteins and stress-tolerant biomolecules. Overall, this study broadens our understanding of the cross-generic dependencies prevailing in a bacterial community. The insights of cooperation can be further exploited to rationally design a synthetic consortium for remediation of multiple pollutants. This study led us to propose the model proposing interactions amongst the three members of SCP consortium in azo dye degradation.

## Methods

### Strain specifics and decolorization assay

Three different bacterial strains used in this study are *Stenotrophomonas acidaminiphila* APG1, *Pseudomonas stutzeri* APG2 and *Cellulomonas* sp. APG4. These strains were isolated from a mixed bacterial culture, enriched for the decolorization of mono-azo copper-complexed dye, Reactive Blue 28 (RB28) from the sediment of the Alang ship recycling yard, Gujarat, India. The decolorization by these three isolates was optimized in the presence of glycerol as co-substrate. Glycerol being an in-expensive substrate was employed as a co-substrate for the reduction of RB28 as the azo dye cannot be utilized as a sole source of carbon and energy. For decolorization studies, the three isolates were grown in Luria Bertani Broth for overnight at 37 °C under the shaking condition (i.e., 150 rpm). The cells were harvested, washed thrice with sterile distilled water and re-suspended in the same. The cell suspension of all the three cultures were then mixed at equal cell density (660 nm) to be used as inoculum; for decolorization experiments the fresh inoculum so developed was inoculated to achieve an initial cell density of ~ 0.05 (660 nm). The decolorization of RB28 was conducted in sugar tubes containing 20 mL of sterile Bushnell Haas Broth (g L^− 1^: MgSO_4_, 0.2; K_2_HPO_4_, 1.0; CaCl_2_, 0.02; FeCl_3_, 0.05; NH_4_NO_3_, 1.0; pH of 7.0 ± 0.2 at 25 °C) augmented with 100 mg L^− 1^ of the dye and very low concentrations of glycerol (i.e., 0.1% v/v), followed by its inoculation with the consortium and incubation at 37 °C under the static condition till decolorization. The decolorization efficiency of the consortium SCP was determined by measuring the decrease in the absorbance at λ_max_ (570 nm) of the RB28 dye. The dye removal was represented in terms of percent decolorization of the initial dye concentration at the onset of experiment. Also, the effect of 1 mM of redox mediators like anthraquinone-2,6-disulphonate (AQDS), anthraquinone-2-sulphonate (AQS) and 2-hydroxy-1,4-naphthoquinone (lawsone) on the rate of decolorization was studied. The UV-visible spectroscopic analysis of cell-free supernatant before and after decolorization was also carried out. The three bacterial isolates were otherwise maintained as pure cultures on Luria Bertani agar.

### DNA extraction and genome sequencing

**A**ll isolates were grown in 50 mL Luria Bertani broth and incubated at 37 °C under shaking condition. The protocol recommended by Derive and Greek (2012) was used to isolate genomic DNA from the overnight grown cultures [75]. The quality of DNA was assessed by agarose gel electrophoresis, followed by its quantification using Qubit (Thermo, USA). The genome sequencing library for Illumina was prepared using Nextera™ DNA Flex library preparation kit (Illumina Inc., San Diego, CA, USA). The next-generation sequencing was carried out on the Illumina Miseq platform (Illumina Inc., San Diego, CA, USA) by paired-end (2 × 250 bp) technology using V2 Illumina chemistry.

### Genome assembly and annotation

The obtained paired-end raw-reads were examined for their quality employing FastQC [76]. The sequences were trimmed from 3′ and 5′ ends with cutadapt in order to attain a Q-score of ≥30 [77]. The assembly of the processed reads was carried out using assembler, SPAdes- 3.12.0 [78]. The quality of the assembly was assessed using a tool called QUAST [79]. The prediction of the sequences coding for tRNAs and rRNAs from the assembled genome was carried out employing tRNAscan-SE and RNAmmer, respectively [80, 81]. The annotation of the genomes was carried out by uploading scaffolds’ file in Rapid Annotations using Subsystems Technology (RAST) [26]. The number of ORFs was predicted by using Prodigal followed by ORF identification by using DIAMOND Blast [82] and HMMscan [83], where NCBI nr database and Pfam database were used as a reference. Additionally, the functional assignments were performed using eggNOG and COG (clusters of orthologous groups) employing OmicsBox software Version 1.2.4 (https://www.biobam.com/omicsbox). The determination of antibiotic resistance of strains was conducted with the help of the Comprehensive Antibiotic Resistance Database (CARD) [29]. The identification of the prophage sequences from the assembled genomes was performed by PHASTER (PHAge Search Tool – Enhanced Release) [84]. The insertion sequence (IS) elements and tandem repeats present within the genome were investigated by ISfinder [85] and Tandem Repeats Finder [60], respectively. The detection of genomic islands was done using two prediction models, i.e., IslandPath-DIMOB and SIGI HMM in IslandViewer 4 [31]. The loci of clustered regularly interspaced short palindromic repeat sequences and Cas proteins were identified using CRISPRCas finder [30]. The prediction of secondary metabolites was conducted using AntiSMASH (antibiotics and secondary metabolite analysis shell) version 3.0 [86]. A BLAST atlas was generated by comparing APG4 and APG1 genome with the APG2 genome as a reference at the GView server (https://server.gview.ca/) [87]. An identity cut-off of 30% was set to include proteins with lower similarity. The circular map for the genome comparisons of the bacterial isolates was generated employing the BLAST Ring Image generator [88]. The genomes were further submitted in GenBank by NCBI Prokaryotic Genomes Automatic Pipeline (PGAP).

### Inter-generic phylogenetic evaluation

The phylogenetic relationship of *Stenotrophomonas acidaminiphila* APG1, *Pseudomonas stutzeri* APG2 and *Cellulomonas* sp. APG4 was analyzed. The 16S rRNA gene sequences retrieved using RNAmmer from draft genomes of all three isolates were subjected to the nucleotide BLAST against the NCBI database. The sequences of the closest type strains were downloaded and aligned against the query sequences in Clustal X [89]. The aligned sequences were trimmed using DAMBE 6 (Data Analysis in Molecular Biology and Evolution) [90] and then imported in the MEGA X (Molecular Evolutionary Genetics Analysis Software) [91]. Tamura-Nei model, in association with gamma distribution, was predicted to be the best substitution model for phylogenetic evaluation of these sequences, and thus, a phylogenetic tree was constructed using this model with the neighbor-joining method [92]. Moreover, in silico DNA-DNA hybridization (ddh) amongst the genomes of the consortium members was conducted using GGDC (genome-to-genome distance calculator 2.1) webserver (http://ggdc.dsmz.de/home.php) with formula 2 [93]. The overall genome relatedness indices (OGRI) were calculated based on the OrthoANI algorithm of an average nucleotide identity (ANI) calculator [94] at EZBiocloud [95].

### Mechanism of glycerol utilization and azo dye degradation

The glycerol utilization by the APG isolates was studied by co-culturing them on sterile Bushnell Haas agar plates supplemented with 0.1% (v/v) of glycerol (glycerol agar plates) followed by incubation at 37 °C for 72 h. To examine the reliance of isolates on each other with respect to utilization of glycerol for growth, they were streaked in the close vicinity on glycerol agar plates. Moreover, the pathways involved in uptake and utilization of glycerol in all the isolates were inferred from their genome annotation data and retrieving the enzyme functions and pathway details from BioCyc database collection [96] and KEGG (Kyoto Encyclopedia of Genes and Genomes) database (https://www.genome.jp/kegg/pathway.html) [97]. In order to discern the mechanism of the azo dye degradation under anoxic and oxidative conditions, the genome of each isolate was manually inspected for the coding sequences of the putative reductases, oxidases and redox-active molecules involved in the various steps of degradation. Besides, the genes associated with chemotaxis and signal transduction, cell motility and degradation of aromatic compounds were also identified in each genome. The circos plot was made employing circlize package in R [98].

## Supplementary Information

**Additional file 1.**

**Additional file 2.**

**Additional file 3.**

**Additional file 4.**

**Additional file 5.**

**Additional file 6.**

**Additional file 7.**

**Additional file 8.**

**Additional file 9.**

**Additional file 10.**

**Additional file 11.**

**Additional file 12.**

**Additional file 13.**

**Additional file 14.**

**Additional file 15.**

## Data Availability

The draft-genome sequences of all three isolates studied has been deposited as whole-genome shotgun project at GenBank (https://www.ncbi.nlm.nih.gov/) under the accession number JAACYG000000000 (*Stenotrophomonas acidaminiphila* APG1), JAACYH000000000 (*Pseudomonas stutzeri* APG2) and JAACYI000000000 (*Cellulomonas* sp. APG4).
