# Supplementary material for: Genome analysis to decipher syntrophy in the bacterial consortium ‘SCP’ for azo dye degradation
Source: BMC Microbiol. 2021 Jun 11;21:177. doi: 10.1186/s12866-021-02236-9 (PMC8194134; doi:10.1186/s12866-021-02236-9)
Supplement: Supplementary file 1 — Additional file 1. [file 12866_2021_2236_MOESM1_ESM.docx]

**Genome analysis to decipher syntrophy in the bacterial consortium ‘SCP’ for azo dye degradation**

Sandhya Nanjani^a^, Dhiraj Paul^b^ and Hareshkumar Keharia^a^

*^a^Post Graduate Department of Biosciences, UGC Centre of Advanced Study, Sardar Patel University, Gujarat, India*

*^b^Microbial Culture Collection, National Centre for Microbial Resource, National Centre for Cell Science, Savitribai Phule University of Pune Campus, Pune, India*

***Corresponding Author:**

Hareshkumar Keharia

Professor

**Address for correspondence:**

Post Graduate Department of Biosciences,

UGC-Centre of Advanced Study,

Sardar Patel University, Satellite Campus,

Vadtal Road, Bakrol 388 315, Anand, Gujarat.

E-Mail address: haresh970@gmail.com

Phone Number: +91 9924136347


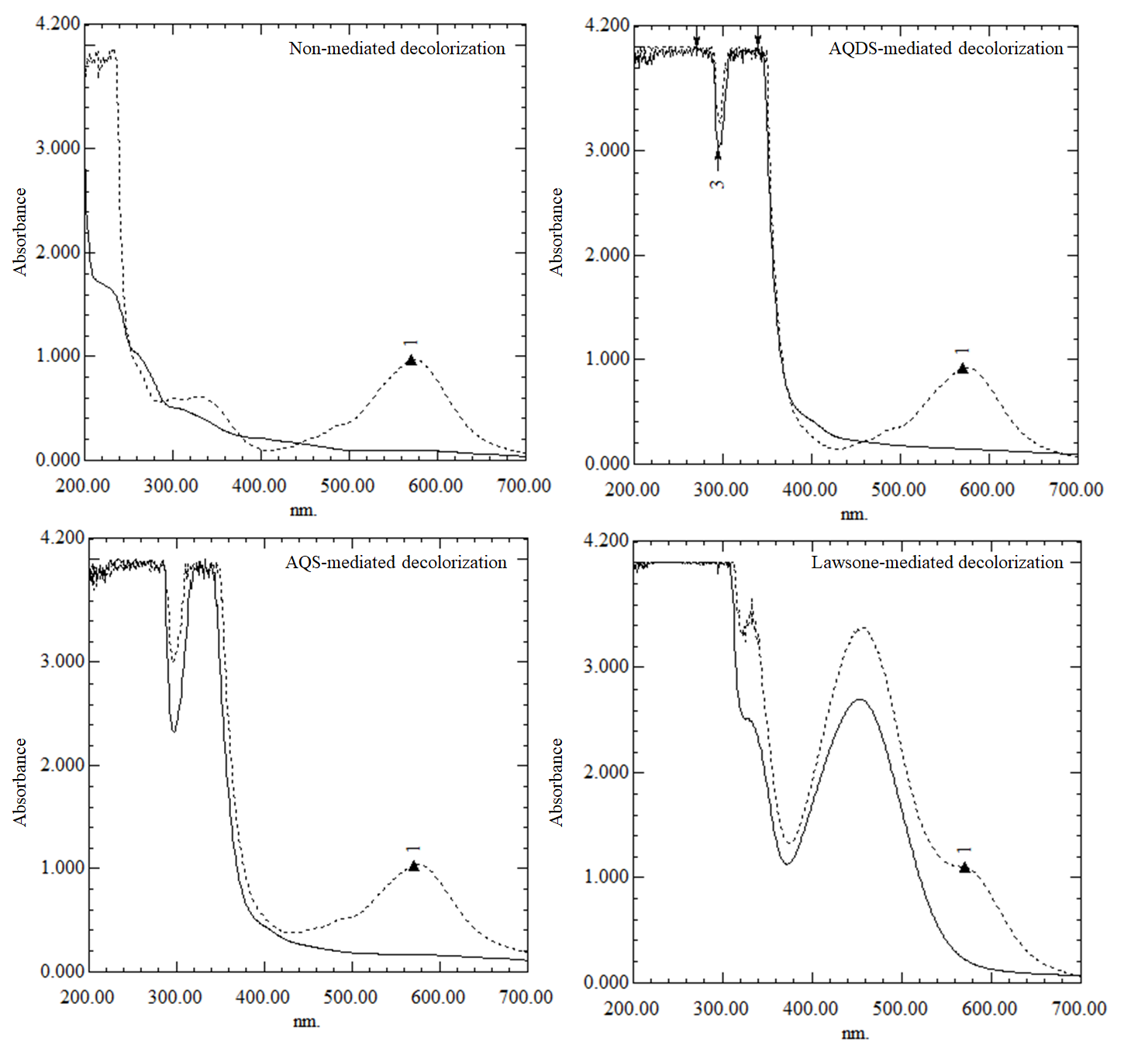


**Additional file 1: Figure S1.** UV-visible spectrum of cell-free supernatant obtained before and after decolorization wherein broken line represents spectra of 0 h, and the entire line represents the spectra after complete decolorization.
