# Supplementary material for: Genome analysis to decipher syntrophy in the bacterial consortium ‘SCP’ for azo dye degradation
Source: BMC Microbiol. 2021 Jun 11;21:177. doi: 10.1186/s12866-021-02236-9 (PMC8194134; doi:10.1186/s12866-021-02236-9)
Supplement: Supplementary file 2 — Additional file 2. [file 12866_2021_2236_MOESM2_ESM.docx]

**
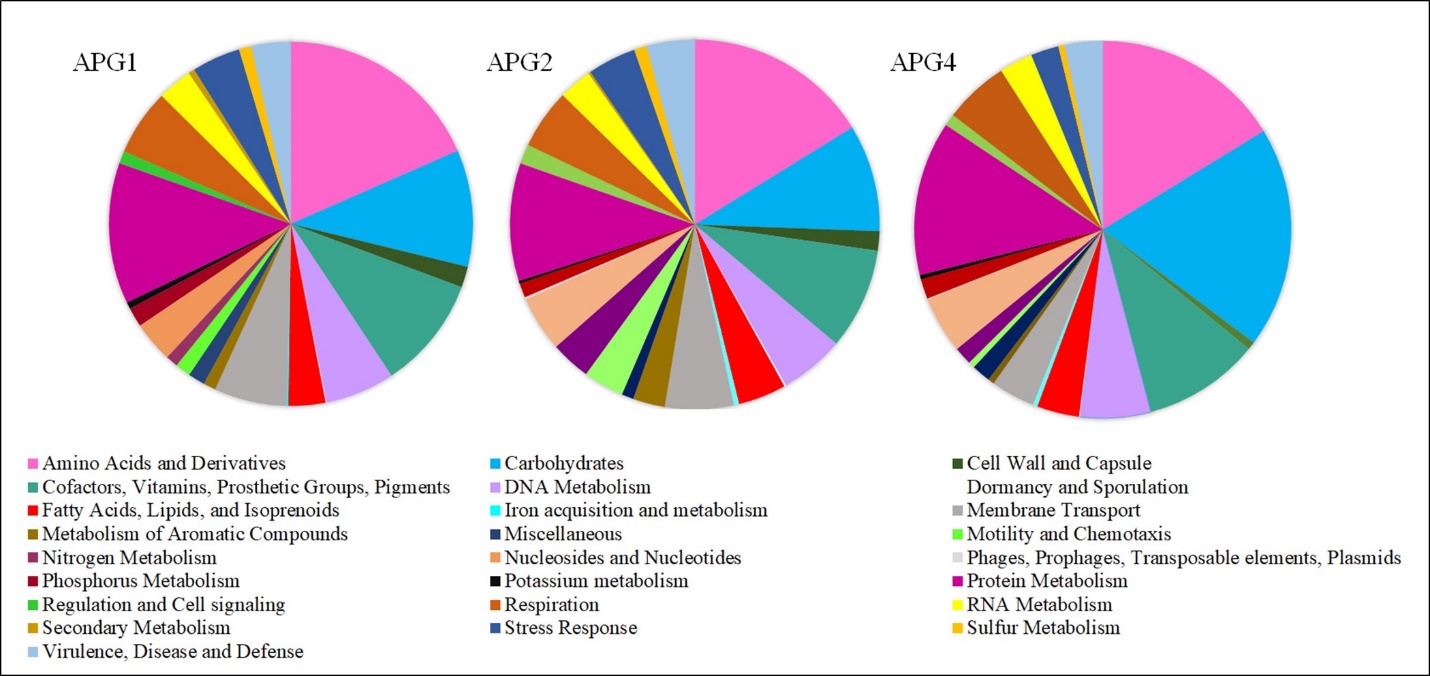
**

**Additional file 2: Figure S2** An overview of subsystem categories distributions obtained from annotation of draft genome sequences of all APG isolates at the RAST (Rapid Annotation System Technology) server.
