# Supplementary material for: Genome analysis to decipher syntrophy in the bacterial consortium ‘SCP’ for azo dye degradation
Source: BMC Microbiol. 2021 Jun 11;21:177. doi: 10.1186/s12866-021-02236-9 (PMC8194134; doi:10.1186/s12866-021-02236-9)
Supplement: Supplementary file 3 — Additional file 3. [file 12866_2021_2236_MOESM3_ESM.docx]

**Additional file 3: Table S1.** Details of genomic islands detected in APG1, APG2 and APG4 genome using IslandPath DIMOB in IslandViewer 4 against the genome *Stenotrophomonas acidaminiphila* SUNEO, *Pseudomonas stutzeri* A1501 and *Cellulomonas flavigena* DSM 20109, respectively.

| **No.** | **APG1** | | **APG2** | | **APG4** | |
| --- | --- | --- | --- | --- | --- | --- |
|  | **Genomic island** | **Length** | **Genomic island** | **Length** | **Genomic island** | **Length** |
| 1 | 17789-29029 | 11240 | 4443-26447 | 22004 | 58-8957 | 8899 |
| 2 | 231242-238042 | 6800 | 302694-307069 | 4375 | 17749-22356 | 4607 |
| 3 | 347225-353628 | 6403 | 435163-440254 | 5091 | 137917-218506 | 80589 |
| 4 | 361585-367496 | 5911 | 450066-454461 | 4395 | 314107-321484 | 7377 |
| 5 | 371971-377102 | 5131 | 509134-537970 | 28836 | 384353-409647 | 25294 |
| 6 | 469189-495547 | 26358 | 755567-764456 | 8889 | 457991-463595 | 5604 |
| 7 | 497860-522572 | 24712 | 775871-832261 | 56390 | 514437-539551 | 25114 |
| 8 | 499692-540712 | 41020 | 925171-935702 | 10531 | 517323-541187 | 23864 |
| 9 | 692905-698470 | 5565 | 996523-1002835 | 6312 | 702465-706834 | 4369 |
| 10 | 699720-707246 | 7526 | 1305832-1327004 | 21172 | 1362600-1383103 | 20503 |
| 11 | 1062045-1076860 | 14815 | 1339850-1352304 | 12454 | 1830537-1885751 | 55214 |
| 12 | 1367270-1467053 | 99783 | 1391384-1405466 | 14082 | 2304956-2320629 | 15673 |
| 13 | 1594760-1601258 | 6498 | 1565876-1589781 | 23905 | 2322638-2326801 | 4163 |
| 14 | 1712506-1724781 | 12275 | 1596290-1605652 | 9362 | 2338002-2343659 | 5657 |
| 15 | 1725314-1735276 | 9962 | 1597512-1606287 | 8775 | 2963320-2973500 | 10180 |
| 16 | 1741884-1749737 | 7853 | 1610228-1620233 | 10005 | 3088581-3133277 | 44696 |
| 17 | 1761270-1769325 | 8055 | 1631645-1642986 | 11341 | 3523677-3529737 | 6060 |
| 18 | 1781110-1788608 | 7498 | 2439415-2443599 | 4184 | 3524191-3538908 | 14717 |
| 19 | 2059728-2077534 | 17806 | 2726989-2742014 | 15025 | 3666784-3696005 | 29221 |
| 20 | 2457904-2469753 | 11849 | 3051208-3055931 | 4723 | 3683637-3699785 | 16148 |
| 21 | 2524101-2589445 | 65344 | 3111537-3116521 | 4984 | 3743207-3747505 | 4298 |
| 22 | 2584034-2591092 | 7058 | 3234850-3240187 | 5337 |  |  |
| 23 | 3024515-3035263 | 10748 | 3376197-3382928 | 6731 |  |  |
| 24 | 3040633-3045876 | 5243 | 3478209-3487092 | 8883 |  |  |
| 25 | 3049936-3059280 | 9344 | 3492429-3497519 | 5090 |  |  |
| 26 | 3069793-3074497 | 4704 | 3613113-3620499 | 7386 |  |  |
| 27 | 3079816-3088510 | 8694 | 3633216-3686900 | 53684 |  |  |
| 28 | 3091953-3109997 | 18044 | 3694909-3722103 | 27194 |  |  |
| 29 | 3118854-3124481 | 5627 | 3721199-3727888 | 6689 |  |  |
| 30 | 3318990-3323189 | 4199 | 3974604-3980322 | 5718 |  |  |
| 31 | 3504514-3509267 | 4753 | 4109118-4137529 | 28411 |  |  |
| 32 | 3523258-3530014 | 6756 | 4415479-4421574 | 6095 |  |  |
| 33 | 3600694-3606242 | 5548 | 4468938-4480975 | 12037 |  |  |
| 34 | 3812846-3822084 | 9238 | 4524215-4531314 | 7099 |  |  |
| 35 | 4077107-4097011 | 19904 | 4531318-4543643 | 12325 |  |  |
| 36 | 4100959-4114505 | 13546 | 4604946-4611221 | 6275 |  |  |
| 37 | 4142830-4155165 | 12335 | 4641635-4661347 | 19712 |  |  |
| 38 | 4172142-4177774 | 5632 | 4641888-4752537 | 110649 |  |  |
| **Total** |  | **553777** |  | **616150** |  | **412247** |
