# Supplementary material for: Genome analysis to decipher syntrophy in the bacterial consortium ‘SCP’ for azo dye degradation
Source: BMC Microbiol. 2021 Jun 11;21:177. doi: 10.1186/s12866-021-02236-9 (PMC8194134; doi:10.1186/s12866-021-02236-9)
Supplement: Supplementary file 4 — Additional file 4. [file 12866_2021_2236_MOESM4_ESM.docx]

**Additional file 4: Table S2.** Proteins encoded in genomic islands of the APG genomes obtained by prediction in IslandViewer 4.

| **S. No.** | **Proteins encoded by sequences in genomic islands** | **APG1** | **APG2** | **APG4** |
| --- | --- | --- | --- | --- |
| 1 | (Fe-S)-binding protein | 1 | 0 | 1 |
| 2 | 1,2-dihydroxycyclohexa-3,5-diene-1-carboxylate dehydrogenase (EC 1.3.1.25) | 0 | 1 | 0 |
| 3 | 16S rRNA (adenine(1518)-N(6)/adenine(1519)-N(6))- dimethyltransferase RsmA | 0 | 0 | 1 |
| 4 | 2,3-butanediol dehydrogenase | 1 | 0 | 0 |
| 5 | 2,3-butanediol dehydrogenase, R-alcohol forming, (R)- and (S)-acetoin-specific (EC 1.1.1.4) | 0 | 1 | 0 |
| 6 | 2,3-dihydroxy-p-cumate-3,4-dioxygenase (CmtC) | 0 | 1 | 0 |
| 7 | 2-hydroxy-3-oxopropionate reductase (EC 1.1.1.60) | 0 | 1 | 0 |
| 8 | 2-hydroxyhexa-2,4-dienoate hydratase (EC 4.2.1.132) | 0 | 1 | 0 |
| 9 | 2-hydroxymuconic semialdehyde hydrolase (EC 3.7.1.9) | 0 | 1 | 0 |
| 10 | 2-keto-3-deoxy-D-arabino-heptulosonate-7- phosphate synthase I alpha (EC 2.5.1.54) | 0 | 1 | 0 |
| 11 | 3'(2'),5'-bisphosphate nucleotidase CysQ | 0 | 0 | 1 |
| 12 | 30S ribosomal protein S18 | 0 | 0 | 1 |
| 13 | 30S ribosomal protein S6 | 0 | 0 | 1 |
| 14 | 30S ribosomal protein S6--L-glutamate ligase | 1 | 0 | 0 |
| 15 | 3-demethylubiquinone-9 3-methyltransferase | 0 | 2 | 0 |
| 16 | 3-deoxy-D-manno-octulosonic acid kinase | 1 | 0 | 0 |
| 17 | 3-oxoacyl-[acyl-carrier protein] reductase (EC 1.1.1.100) | 0 | 1 | 0 |
| 18 | 4-(cytidine 5'-diphospho)-2-C-methyl-D-erythritol kinase | 0 | 0 | 1 |
| 19 | 4-hydroxy-2-oxovalerate aldolase (EC 4.1.3.39) | 0 | 1 | 0 |
| 20 | 4-oxalocrotonate decarboxylase (EC 4.1.1.77) | 0 | 1 | 0 |
| 21 | 4-oxalocrotonate tautomerase( EC:5.3.2.- ) | 0 | 1 | 0 |
| 22 | 5-methyltetrahydrofolate--homocysteine methyltransferase (EC 2.1.1.13) | 0 | 1 | 0 |
| 23 | A/G-specific adenine glycosylase (EC 3.2.2.-) | 0 | 1 | 0 |
| 24 | AAA family ATPase | 5 | 0 | 4 |
| 25 | ABC transporter ATP-binding protein | 1 | 0 | 4 |
| 26 | ABC transporter permease/subunit | 3 | 0 | 4 |
| 27 | ABC transporter substrate-binding protein | 3 | 0 | 0 |
| 28 | ABC-F family ATP-binding cassette domain-containing protein | 0 | 0 | 1 |
| 29 | abortive infection family protein | 1 | 0 | 0 |
| 30 | Acetaldehyde dehydrogenase, acetylating, (EC 1.2.1.10) in gene cluster for degradation of phenols, c | 0 | 1 | 0 |
| 31 | acetate/propionate family kinase | 1 | 0 | 0 |
| 32 | acetoacetyl-CoA reductase | 1 | 0 | 0 |
| 33 | Acetoin dehydrogenase E1 component alpha/beta-subunit (EC 2.3.1.190) | 0 | 2 | 0 |
| 34 | Acetyl-CoA acetyltransferase (EC 2.3.1.9) | 0 | 1 | 0 |
| 35 | Acetyltransferase | 0 | 1 | 0 |
| 36 | Acyl-CoA dehydrogenase 2 [fadN-fadA-fadE operon] (EC 1.3.8.7) | 0 | 1 | 0 |
| 37 | acyl-CoA desaturase | 1 | 0 | 0 |
| 38 | acyltransferase | 1 | 0 | 0 |
| 39 | adenine methyltransferase | 1 | 0 | 0 |
| 40 | Adenine-specific DNA methyltransferase | 0 | 1 | 0 |
| 41 | Aerotaxis sensor receptor protein | 0 | 1 | 0 |
| 42 | Alcohol dehydrogenase (EC 1.1.1.1) | 0 | 1 | 0 |
| 43 | Alkyl hydroperoxide reductase protein F | 0 | 1 | 0 |
| 44 | AlpA family phage regulatory protein | 3 | 0 | 0 |
| 45 | Alpha,alpha-trehalose-phosphate synthase [UDP-forming] (EC 2.4.1.15) | 0 | 1 | 0 |
| 46 | alpha/beta fold hydrolase | 2 | 0 | 0 |
| 47 | alpha/beta hydrolase | 1 | 0 | 2 |
| 48 | Alpha-D-ribose 1-methylphosphonate 5-phosphate C-P lyase (EC 4.7.1.1) | 0 | 1 | 0 |
| 49 | Alpha-D-ribose 1-methylphosphonate 5-triphosphate diphosphatase (EC 3.6.1.63) | 0 | 1 | 0 |
| 50 | Alpha-D-ribose 1-methylphosphonate 5-triphosphate synthase subunit PhnG-L (EC 2.7.8.37) | 0 | 4 | 0 |
| 51 | Amino acid ABC transporter ATP-binding protein | 0 | 0 | 1 |
| 52 | Amino acid ABC transporter permease | 0 | 0 | 1 |
| 53 | Amino acid ABC transporter substrate-binding protein | 0 | 0 | 2 |
| 54 | Amino acid transporters | 0 | 1 | 0 |
| 55 | Aminocarboxymuconate-semialdehyde decarboxylase( EC:4.1.1.45 ) | 0 | 1 | 0 |
| 56 | Ammonium transporter | 0 | 1 | 0 |
| 57 | Anhydro-N-acetylmuramic acid kinase (EC 2.7.1.170) | 0 | 1 | 0 |
| 58 | ankyrin repeat domain-containing protein | 3 | 0 | 0 |
| 59 | Anti-sigma factor | 0 | 0 | 1 |
| 60 | Apolipoprotein N-acyltransferase | 0 | 0 | 2 |
| 61 | ArdC family protein | 1 | 0 | 0 |
| 62 | Arginase family protein | 0 | 0 | 1 |
| 63 | arginine/agmatine antiporter | 1 | 0 | 0 |
| 64 | asparagine synthase | 1 | 0 | 0 |
| 65 | Aspartyl-tRNA synthetase (EC 6.1.1.12) @ Aspartyl-tRNA(Asn) synthetase (EC 6.1.1.23) | 0 | 1 | 0 |
| 66 | ATP/GTP-binding protein | 0 | 1 | 0 |
| 67 | ATPase required for both assembly of type IV secretion complex and secretion of T-DNA complex, VirB1/VirB4 | 0 | 2 | 0 |
| **S. No.** | **Proteins encoded by sequences in genomic islands** | **APG1** | **APG2** | **APG4** |
| 68 | ATP-binding domain-containing protein | 0 | 0 | 1 |
| 69 | ATP-binding protein | 5 | 0 | 3 |
| 70 | ATP-dependent helicase | 1 | 0 | 0 |
| 71 | ATP-dependent hsl protease ATP-binding subunit HslU | 0 | 1 | 0 |
| 72 | ATP-dependent protease subunit HslV (EC 3.4.25.2) | 0 | 1 | 0 |
| 73 | Atxe2 family lasso peptide isopeptidase | 1 | 0 | 0 |
| 74 | benenodin family lasso peptide | 1 | 0 | 0 |
| 75 | beta-lactamase family protein | 2 | 0 | 0 |
| 76 | beta-phosphoglucomutase family hydrolase | 0 | 0 | 1 |
| 77 | bifunctional copper resistance protein CopD/cytochrome c oxidase assembly protein | 0 | 0 | 1 |
| 78 | bifunctional enoyl-CoA hydratase/phosphate acetyltransferase | 1 | 0 | 0 |
| 79 | Biotin operon repressor / Biotin--protein ligase (EC 6.3.4.9)(EC 6.3.4.10)(EC 6.3.4.11)(EC 6.3.4.15) | 0 | 1 | 0 |
| 80 | Bll5738 protein | 0 | 1 | 0 |
| 81 | Bores hole in peptidoglycan layer allowing type IV secretion complex assembly to occur (VirB1) | 0 | 1 | 0 |
| 82 | cadmium transporter | 0 | 0 | 2 |
| 83 | cadmium-translocating P-type ATPase | 1 | 0 | 1 |
| 84 | calcium-binding protein | 1 | 0 | 0 |
| 85 | Candidate type III effector Hop protein | 0 | 1 | 0 |
| 86 | carbohydrate ABC transporter permease | 0 | 0 | 1 |
| 87 | carbohydrate ABC transporter substrate-binding protein | 0 | 0 | 1 |
| 88 | carbon storage regulator CsrA | 0 | 0 | 1 |
| 89 | Carbonic anhydrase, beta class (EC 4.2.1.1) | 0 | 1 | 0 |
| 90 | cation transporter | 3 | 0 | 1 |
| 91 | cbb3-type cytochrome oxidase assembly protein CcoS | 1 | 0 | 0 |
| 92 | CBS domain-containing protein | 1 | 0 | 0 |
| 93 | CCA tRNA nucleotidyltransferase | 0 | 0 | 1 |
| 94 | Cell division protein FtsH | 0 | 1 | 0 |
| 95 | cell division protein SepF | 0 | 0 | 1 |
| 96 | chemotaxis protein CheA/CheW | 0 | 0 | 2 |
| 97 | Chromosome (plasmid) partitioning protein ParA/ParB | 0 | 2 | 0 |
| 98 | Chromosome partition protein MukB | 0 | 1 | 0 |
| 99 | class I SAM-dependent DNA methyltransferase | 0 | 0 | 2 |
| 100 | class I SAM-dependent methyltransferase | 4 | 0 | 0 |
| 101 | Cobalt/zinc/cadmium efflux RND transporter, membrane fusion protein, CzcB family | 0 | 1 | 0 |
| 102 | Cobalt-zinc-cadmium resistance protein CzcA; Cation efflux system protein CusA | 0 | 2 | 0 |
| 103 | Cobalt-zinc-cadmium resistance protein CzcD | 0 | 1 | 0 |
| 104 | cointegrate resolution protein T | 1 | 0 | 0 |
| 105 | cold shock domain-containing protein | 0 | 0 | 1 |
| 106 | Cold shock protein of CSP family | 0 | 1 | 0 |
| 107 | conjugal transfer protein TraD/TraG | 3 | 0 | 0 |
| 108 | CopG family transcriptional regulator | 1 | 1 | 0 |
| 109 | CopK family periplasmic copper-binding protein | 1 | 0 | 0 |
| 110 | copper homeostasis membrane protein CopD | 1 | 0 | 0 |
| 111 | copper homeostasis protein CutC | 1 | 0 | 0 |
| 112 | Copper resistance protein B | 1 | 2 | 0 |
| 113 | copper resistance protein CopC | 0 | 0 | 1 |
| 114 | copper resistance system multicopper oxidase | 1 | 2 | 1 |
| 115 | Copper sensory histidine kinase CusS | 0 | 1 | 0 |
| 116 | Copper-sensing two-component system response regulator CusR | 0 | 1 | 0 |
| 117 | copper-translocating P-type ATPase | 0 | 0 | 1 |
| 118 | coproporphyrinogen III oxidase | 1 | 0 | 0 |
| 119 | cryptochrome/photolyase family protein | 1 | 0 | 0 |
| 120 | c-type cytochrome | 2 | 0 | 0 |
| 121 | c-type cytochrome biogenesis protein CcsB | 0 | 0 | 1 |
| 122 | CusA/CzcA family heavy metal efflux RND transporter | 1 | 0 | 0 |
| 123 | Cyanate hydratase (EC 4.2.1.104) | 0 | 1 | 0 |
| 124 | cystathionine gamma-synthase | 1 | 0 | 0 |
| 125 | cytochrome c | 1 | 0 | 0 |
| 126 | cytochrome c biogenesis protein CcdA | 0 | 0 | 2 |
| 127 | cytochrome c biogenesis protein ResB | 0 | 0 | 1 |
| 128 | Cytochrome c family protein | 0 | 2 | 0 |
| 129 | cytochrome c oxidase assembly protein | 0 | 0 | 1 |
| 130 | Cytochrome c oxidase polypeptide I/ II/ III(EC 1.9.3.1) | 0 | 3 | 0 |
| 131 | Cytochrome c-type biogenesis protein DsbD, protein-disulfide reductase (EC 1.8.1.8) | 0 | 1 | 0 |
| 132 | Cytochrome oxidase biogenesis protein Cox11-CtaG, copper delivery to Cox1 | 0 | 1 | 0 |
| 133 | cytotoxic translational repressor of toxin-antitoxin stability system | 0 | 0 | 1 |
| 134 | dCTP deaminase | 0 | 0 | 1 |
| 135 | DDE-type integrase/transposase/recombinase | 0 | 0 | 2 |
| 136 | DEAD/DEAH box helicase | 1 | 1 | 2 |
| 137 | DegT/DnrJ/EryC1/StrS family aminotransferase | 1 | 0 | 0 |
| 138 | D-glycerate 2-kinase (EC 2.7.1.165) | 0 | 1 | 0 |
| 139 | Diacylglycerol kinase | 1 | 0 | 0 |
| 140 | Diadenosine tetraphosphate (Ap4A) hydrolase and other HIT family hydrolases | 0 | 1 | 0 |
| **S. No.** | **Proteins encoded by sequences in genomic islands** | **APG1** | **APG2** | **APG4** |
| 141 | diguanylate cyclase/phosphodiesterase (GGDEF & EAL domains) with PAS/PAC sensor(s) | 0 | 1 | 0 |
| 142 | dihydrofolate reductase | 0 | 0 | 1 |
| 143 | DinB family protein | 0 | 0 | 1 |
| 144 | DMT family transporter | 0 | 0 | 1 |
| 145 | DNA (cytosine-5-)-methyltransferase | 1 | 0 | 0 |
| 146 | DNA cytosine methyltransferase | 1 | 0 | 0 |
| 147 | DNA ligase D | 1 | 0 | 0 |
| 148 | DNA mismatch repair protein MutS | 1 | 0 | 0 |
| 149 | DNA polymerase III subunit gamma/tau | 1 | 0 | 0 |
| 150 | DNA polymerase Y family protein | 1 | 0 | 0 |
| 151 | DNA protection during starvation protein | 0 | 1 | 0 |
| 152 | DNA repair protein RadC | 3 | 0 | 0 |
| 153 | DNA repair protein RecN | 1 | 0 | 0 |
| 154 | DNA topoisomerase III | 1 | 0 | 0 |
| 155 | DNA-3-methyladenine glycosylase | 1 | 0 | 0 |
| 156 | DNA-binding domain-containing protein | 2 | 0 | 0 |
| 157 | DNA-binding heavy metal response regulator | 0 | 2 | 0 |
| 158 | DNA-binding protein | 0 | 1 | 0 |
| 159 | DNA-directed RNA polymerase alpha/beta/beta' subunit (EC 2.7.7.6) | 0 | 3 | 0 |
| 160 | DNA-processing protein DprA | 1 | 0 | 0 |
| 161 | DoxX family protein | 0 | 1 | 0 |
| 162 | DsrE family protein | 1 | 0 | 0 |
| 163 | dTDP-4-amino-4,6-dideoxygalactose transaminase | 1 | 0 | 0 |
| 164 | DUF1156 domain-containing protein | 1 | 0 | 0 |
| 165 | DUF1295 domain-containing protein | 1 | 0 | 0 |
| 166 | DUF1365 domain-containing protein | 1 | 0 | 0 |
| 167 | DUF1738 domain-containing protein | 2 | 0 | 0 |
| 168 | DUF1801 domain-containing protein | 0 | 0 | 1 |
| 169 | DUF2283 domain-containing protein | 0 | 0 | 1 |
| 170 | DUF2293 domain-containing protein | 1 | 0 | 0 |
| 171 | DUF2326 domain-containing protein | 1 | 0 | 0 |
| 172 | DUF2530 domain-containing protein | 0 | 0 | 1 |
| 173 | DUF262 domain-containing protein | 1 | 0 | 0 |
| 174 | DUF2637 domain-containing protein | 0 | 0 | 1 |
| 175 | DUF2857 family protein | 1 | 0 | 0 |
| 176 | DUF2878 domain-containing protein | 1 | 0 | 0 |
| 177 | DUF2892 domain-containing protein | 1 | 0 | 0 |
| 178 | DUF2933 domain-containing protein | 1 | 0 | 0 |
| 179 | DUF2971 domain-containing protein | 1 | 0 | 0 |
| 180 | DUF305 domain-containing protein | 0 | 0 | 3 |
| 181 | DUF3085 domain-containing protein | 3 | 0 | 0 |
| 182 | DUF3105 domain-containing protein | 0 | 0 | 1 |
| 183 | DUF3141 domain-containing protein | 1 | 0 | 0 |
| 184 | DUF3158 family protein | 1 | 0 | 0 |
| 185 | DUF3275 family protein | 2 | 0 | 0 |
| 186 | DUF3320 domain-containing protein | 0 | 0 | 1 |
| 187 | DUF3577 domain-containing protein | 2 | 0 | 0 |
| 188 | DUF3606 domain-containing protein | 1 | 0 | 0 |
| 189 | DUF3742 family protein | 2 | 0 | 0 |
| 190 | DUF3800 domain-containing protein | 0 | 0 | 1 |
| 191 | DUF3883 domain-containing protein | 1 | 0 | 0 |
| 192 | DUF3987 domain-containing protein | 2 | 0 | 0 |
| 193 | DUF4105 domain-containing protein | 1 | 0 | 0 |
| 194 | DUF411 domain-containing protein | 1 | 0 | 0 |
| 195 | DUF4129 domain-containing protein | 0 | 0 | 1 |
| 196 | DUF4235 domain-containing protein | 0 | 0 | 1 |
| 197 | DUF4333 domain-containing protein | 0 | 0 | 1 |
| 198 | DUF4338 domain-containing protein | 1 | 0 | 0 |
| 199 | DUF4389 domain-containing protein | 0 | 0 | 1 |
| 200 | DUF4411 family protein | 0 | 0 | 1 |
| 201 | DUF58 domain-containing protein | 0 | 0 | 1 |
| 202 | DUF853 family protein | 1 | 0 | 0 |
| 203 | DUF932 domain-containing protein | 1 | 0 | 0 |
| 204 | EamA family transporter | 0 | 0 | 1 |
| 205 | Eco29kI family restriction endonuclease | 1 | 0 | 0 |
| 206 | efflux RND transporter periplasmic adaptor subunit | 3 | 0 | 0 |
| 207 | efflux RND transporter permease subunit | 1 | 0 | 0 |
| 208 | efflux transporter outer membrane subunit | 2 | 0 | 0 |
| 209 | endonuclease | 1 | 0 | 0 |
| 210 | Epoxide hydrolase (EC 3.3.2.9) | 0 | 1 | 0 |
| 211 | error-prone DNA polymerase | 1 | 0 | 0 |
| 212 | Error-prone, lesion bypass DNA polymerase V (UmuC) | 0 | 1 | 0 |
| 213 | EscU/YscU/HrcU family type III secretion system export apparatus switch protein | 0 | 0 | 1 |
| **S. No.** | **Proteins encoded by sequences in genomic islands** | **APG1** | **APG2** | **APG4** |
| 214 | excisionase family DNA-binding protein | 0 | 0 | 3 |
| 215 | FAD-dependent oxidoreductase | 1 | 0 | 0 |
| 216 | family 78 glycoside hydrolase catalytic domain | 0 | 0 | 1 |
| 217 | fasciclin domain-containing protein | 1 | 0 | 0 |
| 218 | Ferredoxin, 2Fe-2S | 0 | 1 | 0 |
| 219 | ferric iron uptake transcriptional regulator | 1 | 0 | 0 |
| 220 | FHIPEP family type III secretion protein | 0 | 0 | 1 |
| 221 | Fic family protein | 0 | 0 | 1 |
| 222 | FIG001341: Probable Fe(2+)-trafficking protein YggX | 0 | 1 | 0 |
| 223 | FIG051360: Periplasmic protein TonB, links inner and outer membranes | 0 | 1 | 0 |
| 224 | flagellar basal body-associated FliL family protein | 0 | 0 | 1 |
| 225 | Flagellar basal-body rod protein FlgB | 0 | 1 | 0 |
| 226 | flagellar biosynthesis protein FliQ/FliR | 0 | 0 | 2 |
| 227 | flagellar type III secretion system pore protein FliP | 0 | 0 | 1 |
| 228 | FliO/MopB family protein | 0 | 0 | 1 |
| 229 | FRG domain-containing protein | 0 | 0 | 1 |
| 230 | FtsX-like permease family protein | 0 | 0 | 1 |
| 231 | GDP-L-fucose synthetase (EC 1.1.1.271) | 0 | 1 | 0 |
| 232 | GDP-mannose 4,6-dehydratase (EC 4.2.1.47) | 0 | 1 | 0 |
| 233 | GDP-mannose mannosyl hydrolase | 0 | 1 | 0 |
| 234 | GGDEF domain protein | 1 | 1 | 0 |
| 235 | GIY-YIG nuclease family protein | 1 | 0 | 0 |
| 236 | Glucoamylase (EC 3.2.1.3) | 0 | 1 | 0 |
| 237 | Glucose-methanol-choline (GMC) oxidoreductase:NAD binding site | 0 | 1 | 0 |
| 238 | Glutamate--UDP-2-acetamido-2-deoxy-D-ribohex-3- uluronic acid aminotransferase (PLP cofactor) (EC 2. | 0 | 1 | 0 |
| 239 | glutaredoxin | 0 | 0 | 1 |
| 240 | glutathione S-transferase family protein | 1 | 0 | 0 |
| 241 | Glycerate kinase (EC 2.7.1.31) | 0 | 1 | 0 |
| 242 | glycoside hydrolase family 2 protein | 0 | 0 | 1 |
| 243 | glycosyltransferase | 2 | 0 | 0 |
| 244 | glycosyltransferase family 4 protein | 1 | 0 | 2 |
| 245 | Glyoxylate carboligase (EC 4.1.1.47) | 0 | 1 | 0 |
| 246 | GNAT family N-acetyltransferase | 1 | 0 | 1 |
| 247 | GTP cyclohydrolase I | 1 | 0 | 0 |
| 248 | GTPase | 3 | 0 | 0 |
| 249 | HAMP domain-containing protein | 0 | 0 | 1 |
| 250 | heat shock protein DnaJ domain protein | 0 | 1 | 0 |
| 251 | heavy metal response regulator transcription factor | 1 | 0 | 0 |
| 252 | Heavy metal RND efflux outer membrane protein, CzcC family | 0 | 3 | 0 |
| 253 | heavy metal sensor histidine kinase | 1 | 1 | 0 |
| 254 | heavy metal translocating P-type ATPase | 2 | 0 | 3 |
| 255 | heavy metal-responsive transcriptional regulator | 0 | 0 | 2 |
| 256 | heavy-metal-associated domain-containing protein | 0 | 0 | 1 |
| 257 | Helicase, C-terminal:Type III restriction enzyme, res subunit:DEAD/DEAH box helicase, N-terminal | 0 | 1 | 0 |
| 258 | helix-turn-helix domain-containing protein | 5 | 0 | 6 |
| 259 | helix-turn-helix transcriptional regulator | 5 | 0 | 4 |
| 260 | histidinol-phosphate transaminase | 0 | 0 | 1 |
| 261 | HlyD family efflux transporter periplasmic adaptor subunit | 1 | 0 | 0 |
| 262 | HlyD family type I secretion periplasmic adaptor subunit | 1 | 0 | 0 |
| 263 | HNH endonuclease | 4 | 0 | 1 |
| 264 | H-NS histone family protein | 1 | 0 | 0 |
| 265 | HORMA domain containing protein | 1 | 0 | 0 |
| 266 | hydrolase or metal-binding protein | 2 | 0 | 0 |
| 267 | Hydroxypyruvate isomerase (EC 5.3.1.22) | 0 | 1 | 0 |
| 268 | hypothetical plasmid protein | 0 | 1 | 0 |
| 269 | Hypothetical protein involved in heavy metal export | 0 | 1 | 0 |
| 270 | ImmA/IrrE family metallo-endopeptidase | 0 | 0 | 1 |
| 271 | Inner membrane protein forms channel for type IV secretion of T-DNA complex, VirB8 | 0 | 1 | 0 |
| 272 | Inner membrane protein of type IV secretion of T-DNA complex, TonB-like, VirB10 | 0 | 1 | 0 |
| 273 | Inner membrane protein of type IV secretion of T-DNA complex, VirB6 | 0 | 1 | 0 |
| 274 | Inner membrane protein YohC | 0 | 1 | 0 |
| 275 | inositol-3-phosphate synthase | 0 | 0 | 1 |
| 276 | integrase | 5 | 0 | 0 |
| 277 | integrase arm-type DNA-binding domain-containing protein | 3 | 0 | 0 |
| 278 | integrase family protein | 1 | 0 | 0 |
| 279 | integrating conjugative element protein | 3 | 0 | 0 |
| 280 | iron ABC transporter permease | 0 | 0 | 1 |
| 281 | Iron-sulfur cluster insertion protein ErpA | 0 | 1 | 0 |
| 282 | IS1380 family transposase | 0 | 0 | 1 |
| 283 | IS21 family transposase | 0 | 0 | 2 |
| 284 | IS3 family transposase | 3 | 0 | 4 |
| 285 | IS481 family transposase | 0 | 0 | 1 |
| **S. No.** | **Proteins encoded by sequences in genomic islands** | **APG1** | **APG2** | **APG4** |
| 286 | IS5 family transposase | 0 | 0 | 1 |
| 287 | ISL3 family transposase | 0 | 0 | 1 |
| 288 | Isochorismate pyruvate-lyase (EC 4.-.-.-) | 0 | 1 | 0 |
| 289 | JAB domain-containing protein | 1 | 0 | 0 |
| 290 | KorC protein | 0 | 1 | 0 |
| 291 | Ku protein | 1 | 0 | 0 |
| 292 | LacI family transcriptional regulator | 0 | 0 | 1 |
| 293 | lasso peptide biosynthesis B2 protein | 1 | 0 | 0 |
| 294 | Lead, cadmium, zinc and mercury transporting ATPase (EC 3.6.3.3) (EC 3.6.3.5); Copper-translocating | 0 | 3 | 0 |
| 295 | lipid A deacylase LpxR family protein | 2 | 0 | 0 |
| 296 | lipocalin family protein | 1 | 0 | 0 |
| 297 | lipoprotein signal peptidase | 1 | 0 | 1 |
| 298 | Lsr2 family protein | 0 | 0 | 1 |
| 299 | LSU ribosomal protein L10p - L18p | 0 | 6 | 0 |
| 300 | LSU ribosomal protein L1p-L7p | 0 | 7 | 0 |
| 301 | LSU ribosomal protein L22p-L36p | 0 | 6 | 0 |
| 302 | lysine decarboxylase | 1 | 0 | 0 |
| 303 | LysR family transcriptional regulator | 6 | 0 | 0 |
| 304 | LysR-family transcriptional regulator PtxE, associated with phosphonate utilization | 0 | 1 | 0 |
| 305 | lytic transglycosylase domain-containing protein | 1 | 0 | 0 |
| 306 | M15 family metallopeptidase | 1 | 0 | 0 |
| 307 | Malonate decarboxylase alpha subunit | 0 | 1 | 0 |
| 308 | Mannose-1-phosphate guanylyltransferase (EC 2.7.7.13) / Mannose-6-phosphate isomerase (EC 5.3.1.8) | 0 | 1 | 0 |
| 309 | MATE family efflux transporter | 0 | 0 | 1 |
| 310 | MBL fold metallo-hydrolase | 5 | 0 | 0 |
| 311 | mercuric ion transporter MerT | 1 | 0 | 0 |
| 312 | mercury resistance co-regulator MerD | 1 | 0 | 0 |
| 313 | mercury resistance system periplasmic binding protein MerP | 1 | 0 | 0 |
| 314 | mercury resistance transcriptional regulator MerR | 1 | 0 | 0 |
| 315 | mercury(II) reductase | 1 | 0 | 2 |
| 316 | MerR family transcriptional regulator | 0 | 0 | 1 |
| 317 | metallophosphoesterase | 1 | 0 | 0 |
| 318 | metal-sensitive transcriptional regulator | 0 | 0 | 1 |
| 319 | methyl-accepting chemotaxis protein | 0 | 1 | 3 |
| 320 | MFS transporter | 1 | 0 | 1 |
| 321 | MgtC family protein | 0 | 1 | 0 |
| 322 | Minor pilin of type IV secretion complex, VirB5 | 0 | 1 | 0 |
| 323 | Mll5901 protein | 0 | 2 | 0 |
| 324 | MobA/MobL family protein | 3 | 0 | 0 |
| 325 | Mobile element protein | 0 | 18 | 0 |
| 326 | Mobilization protein MobA @ Conjugal transfer protein TraA | 0 | 1 | 0 |
| 327 | molecular chaperone DnaK | 1 | 0 | 0 |
| 328 | monovalent cation/H(+) antiporter subunit G | 0 | 0 | 1 |
| 329 | MoxR family ATPase | 0 | 0 | 1 |
| 330 | MSHA biogenesis protein MshO-MshP | 0 | 2 | 0 |
| 331 | MSHA pilin protein MshD | 0 | 1 | 0 |
| 332 | multidrug efflux RND transporter permease subunit | 1 | 0 | 0 |
| 333 | N(4)-(beta-N-acetylglucosaminyl)-L-asparaginase | 1 | 0 | 0 |
| 334 | N-6 DNA methylase | 2 | 0 | 0 |
| 335 | Na(+)/H(+) antiporter subunit A-/C/ D/E | 0 | 0 | 4 |
| 336 | N-acetyl-gamma-glutamyl-phosphate reductase (EC 1.2.1.38) | 0 | 1 | 0 |
| 337 | NAD(P)/FAD-dependent oxidoreductase | 1 | 0 | 0 |
| 338 | NAD(P)-binding domain-containing protein | 1 | 0 | 0 |
| 339 | NAD(P)H-binding protein | 0 | 0 | 2 |
| 340 | Nitrate/nitrite transporter | 0 | 1 | 0 |
| 341 | Nitrate/nitrite transporter NarK | 0 | 1 | 0 |
| 342 | nitric-oxide reductase large subunit | 1 | 0 | 0 |
| 343 | nitrogen fixation protein FixH | 1 | 0 | 0 |
| 344 | nitrogen regulation protein NR(I) | 1 | 0 | 0 |
| 345 | Nitrogen regulation protein NR(I), GlnG (=NtrC) | 0 | 1 | 0 |
| 346 | Nitrogen regulation protein NtrB (EC 2.7.13.3) | 0 | 1 | 0 |
| 347 | nucleoid-associated protein | 1 | 0 | 0 |
| 348 | nucleoside hydrolase | 0 | 0 | 1 |
| 349 | nucleotide exchange factor GrpE | 1 | 0 | 0 |
| 350 | nucleotide-binding protein | 1 | 0 | 0 |
| 351 | nucleotidyltransferase | 1 | 0 | 0 |
| 352 | NUDIX hydrolase | 0 | 0 | 1 |
| 353 | OBAP family protein | 1 | 0 | 0 |
| 354 | Oligopeptidase A (EC 3.4.24.70) | 0 | 1 | 0 |
| 355 | OsmC family protein | 1 | 0 | 0 |
| 356 | Outer membrane low permeability porin, OprD family => OccD4/OpdT tyrosine | 0 | 2 | 0 |
| 357 | outer membrane protein assembly factor BamE | 1 | 0 | 0 |
| 358 | Oxidoreductase | 0 | 1 | 0 |
| **S. No.** | **Proteins encoded by sequences in genomic islands** | **APG1** | **APG2** | **APG4** |
| 359 | PaaI family thioesterase | 1 | 0 | 0 |
| 360 | PadR family transcriptional regulator | 0 | 0 | 1 |
| 361 | Pantothenate kinase type III, CoaX-like (EC 2.7.1.33) | 0 | 1 | 0 |
| 362 | ParA family protein | 1 | 1 | 0 |
| 363 | PASTA domain-containing protein | 0 | 0 | 1 |
| 364 | PD-(D/E)XK nuclease family protein | 1 | 0 | 0 |
| 365 | Peptidase, M23/M37 family | 0 | 1 | 0 |
| 366 | Peptide-methionine (S)-S-oxide reductase MsrA (EC 1.8.4.11) | 0 | 2 | 0 |
| 367 | peptidoglycan DD-metalloendopeptidase family protein | 0 | 0 | 1 |
| 368 | Permease of the drug/metabolite transporter (DMT) superfamily | 0 | 1 | 0 |
| 369 | peroxiredoxin family protein | 0 | 0 | 1 |
| 370 | pH regulation protein F | 0 | 0 | 1 |
| 371 | Phage helicase, polymerase, or exonuclease (ACLAME 182) | 0 | 1 | 0 |
| 372 | phage holin family protein | 0 | 0 | 1 |
| 373 | Phage related proteins (integrase, tail tube protein, tail fiber, GpE, GpE', tail sheath monomer) | 0 | 9 | 0 |
| 374 | Phenylpropionate dioxygenase and related ring- hydroxylating dioxygenases, large terminal subunit | 0 | 1 | 0 |
| 375 | Phosphatidylglycerophosphatase A (EC 3.1.3.27) | 0 | 1 | 0 |
| 376 | Phosphoglucomutase (EC 5.4.2.2) | 0 | 1 | 0 |
| 377 | Phosphonate ABC transporter ATP-binding protein PhnC (TC 3.A.1.9.1) | 0 | 1 | 0 |
| 378 | Phosphonate ABC transporter ATP-binding protein PtxA (TC 3.A.1.9.1) | 0 | 1 | 0 |
| 379 | Phosphonate ABC transporter permease protein PhnE (TC 3.A.1.9.1) | 0 | 1 | 0 |
| 380 | Phosphonate ABC transporter permease protein PtxC (TC 3.A.1.9.1) | 0 | 1 | 0 |
| 381 | Phosphonate ABC transporter substrate-binding protein PhnD (TC 3.A.1.9.1) | 0 | 1 | 0 |
| 382 | Phosphonate ABC transporter substrate-binding protein PtxB (TC 3.A.1.9.1) | 0 | 1 | 0 |
| 383 | Phosphonate dehydrogenase (EC 1.20.1.1) | 0 | 1 | 0 |
| 384 | Phosphonates utilization ATP-binding protein PhnK | 0 | 1 | 0 |
| 385 | PIG-L family deacetylase | 0 | 0 | 1 |
| 386 | pilus assembly protein/ PilL | 2 | 0 | 0 |
| 387 | PIN domain-containing protein | 1 | 0 | 0 |
| 388 | Plasmid replication initiator protein | 0 | 1 | 0 |
| 389 | porin | 1 | 0 | 0 |
| 390 | Predicted ATPase (AAA+ superfamily) | 0 | 1 | 0 |
| 391 | Predicted transcriptional regulator LiuR of leucine degradation pathway, MerR family | 0 | 1 | 0 |
| 392 | prepilin-type N-terminal cleavage/methylation domain-containing protein | 5 | 0 | 0 |
| 393 | prevent-host-death protein | 0 | 0 | 1 |
| 394 | Probable Co/Zn/Cd efflux system membrane fusion protein | 0 | 1 | 0 |
| 395 | Probable DEAH ATP-dependent helicase | 0 | 1 | 0 |
| 396 | prolipoprotein diacylglyceryl transferase | 0 | 0 | 2 |
| 397 | protein kinase | 1 | 0 | 0 |
| 398 | protein of unknown function DUF306, Meta and HslJ | 0 | 1 | 0 |
| 399 | Protein SlyX | 0 | 1 | 0 |
| 400 | Protein translocase subunit SecE | 0 | 1 | 0 |
| 401 | Protein translocase subunit SecY | 0 | 1 | 0 |
| 402 | Protein YrdA | 0 | 1 | 0 |
| 403 | protein-disulfide reductase DsbD | 1 | 0 | 0 |
| 404 | Protein-methionine-sulfoxide reductase catalytic subunit MsrP | 0 | 1 | 0 |
| 405 | Protein-methionine-sulfoxide reductase heme-binding subunit MsrQ | 0 | 1 | 0 |
| 406 | Protoporphyrinogen IX oxidase, novel form, HemJ (EC 1.3.-.-) | 0 | 1 | 0 |
| 407 | Putative 5-carboxymethyl-2-hydroxymuconate semialdehyde dehydrogenase oxidoreductase protein (EC 1.2 | 0 | 1 | 0 |
| 408 | Putative dioxygenase | 0 | 1 | 0 |
| 409 | Putative DNA helicase | 0 | 2 | 0 |
| 410 | putative DNA-invertase | 0 | 1 | 0 |
| 411 | Putative formate dehydrogenase oxidoreductase protein | 0 | 1 | 0 |
| 412 | putative hydrolase | 0 | 1 | 0 |
| 413 | putative lipoprotein | 0 | 1 | 0 |
| 414 | putative plasmid stabilization protein | 0 | 1 | 0 |
| 415 | putative protease | 0 | 1 | 0 |
| 416 | putative ribosylglycohydrolase | 0 | 1 | 0 |
| 417 | Putative serine protease | 0 | 1 | 0 |
| 418 | putative site-specific recombinase | 0 | 1 | 0 |
| 419 | pyrroline-5-carboxylate reductase | 1 | 0 | 0 |
| 420 | Pyruvate kinase (EC 2.7.1.40) | 0 | 1 | 0 |
| 421 | Pyruvate kinase family protein | 0 | 1 | 0 |
| 422 | QacE family quaternary ammonium compound efflux SMR transporter | 1 | 0 | 0 |
| 423 | Quinolinate phosphoribosyltransferase [decarboxylating] (EC 2.4.2.19) | 0 | 1 | 0 |
| 424 | Radical SAM domain protein | 0 | 2 | 0 |
| 425 | raqprd family integrative conjugative element protein | 1 | 0 | 0 |
| 426 | RAQPRD family plasmid | 1 | 0 | 0 |
| 427 | Recombinase | 0 | 1 | 0 |
| 428 | recombinase family protein | 0 | 0 | 2 |
| 429 | RecQ family ATP-dependent DNA helicase | 1 | 0 | 0 |
| 430 | redoxin domain-containing protein | 1 | 0 | 0 |
| **S. No.** | **Proteins encoded by sequences in genomic islands** | **APG1** | **APG2** | **APG4** |
| 431 | relaxase domain-containing protein | 1 | 0 | 1 |
| 432 | RES family NAD+ phosphorylase | 2 | 0 | 0 |
| 433 | Resolvase | 0 | 3 | 0 |
| 434 | Resolvase, N-terminal domain | 0 | 1 | 0 |
| 435 | Respiratory nitrate reductase alpha/ beta/ gamma/ delta chain (EC 1.7.99.4) | 0 | 4 | 0 |
| 436 | response regulator | 1 | 0 | 1 |
| 437 | response regulator transcription factor | 2 | 0 | 4 |
| 438 | restriction endonuclease subunit S | 3 | 0 | 0 |
| 439 | rhodanese-like domain-containing protein | 0 | 0 | 1 |
| 440 | RHS repeat-associated core domain-containing protein | 0 | 0 | 1 |
| 441 | ribbon-helix-helix protein, CopG family | 1 | 0 | 0 |
| 442 | ribose-phosphate pyrophosphokinase | 1 | 0 | 0 |
| 443 | RNA helicase | 1 | 0 | 0 |
| 444 | RnfH family protein | 1 | 0 | 0 |
| 445 | ROK family transcriptional regulator | 0 | 0 | 1 |
| 446 | SAM-dependent DNA methyltransferase | 2 | 0 | 0 |
| 447 | SdiA-regulated domain-containing protein | 1 | 0 | 0 |
| 448 | SDR family oxidoreductase | 1 | 0 | 1 |
| 449 | Sea42 | 0 | 1 | 0 |
| 450 | secretion protein HlyD | 1 | 0 | 1 |
| 451 | SHOCT domain-containing protein | 0 | 0 | 1 |
| 452 | sigma-70 family RNA polymerase sigma factor | 1 | 0 | 2 |
| 453 | SIMPL domain-containing protein | 1 | 0 | 0 |
| 454 | single-stranded DNA-binding protein | 1 | 0 | 1 |
| 455 | SirB2 family protein | 1 | 0 | 0 |
| 456 | site-specific DNA-methyltransferase | 1 | 0 | 0 |
| 457 | site-specific integrase | 3 | 0 | 4 |
| 458 | Site-specific recombinase, resolvase family | 0 | 1 | 0 |
| 459 | sodium/glutamate symporter | 1 | 0 | 0 |
| 460 | sodium:proton antiporter | 0 | 0 | 1 |
| 461 | SOS response-associated peptidase | 2 | 0 | 0 |
| 462 | SpoIIE family protein phosphatase | 0 | 0 | 1 |
| 463 | SsrA-binding protein SmpB | 1 | 0 | 0 |
| 464 | SSU ribosomal protein S10p-S14p/ S17p/ S19p | 0 | 7 | 0 |
| 465 | SSU ribosomal protein S3p/ S4p/S5p/S7p/S8p (S3e) | 0 | 5 | 0 |
| 466 | Stability protein StdB | 0 | 1 | 0 |
| 467 | Succinyl-CoA:3-ketoacid-coenzyme A transferase subunit A (EC 2.8.3.5) | 0 | 1 | 0 |
| 468 | Succinyl-CoA:3-ketoacid-coenzyme A transferase subunit B (EC 2.8.3.5) | 0 | 1 | 0 |
| 469 | sugar ABC transporter permease | 0 | 0 | 1 |
| 470 | sugar ABC transporter substrate-binding protein | 0 | 0 | 1 |
| 471 | sulfate adenylyltransferase subunit CysD | 0 | 0 | 1 |
| 472 | sulfite exporter TauE/SafE family protein | 2 | 0 | 0 |
| 473 | sulfonamide-resistant dihydropteroate synthase Sul1 | 1 | 0 | 0 |
| 474 | SulP family inorganic anion transporter | 1 | 0 | 0 |
| 475 | suppressor of fused domain protein | 0 | 0 | 1 |
| 476 | TetR/AcrR family transcriptional regulator | 2 | 0 | 2 |
| 477 | tetratricopeptide repeat protein | 2 | 0 | 0 |
| 478 | thermonuclease family protein | 1 | 0 | 0 |
| 479 | thiamine ABC transporter substrate-binding protein | 0 | 0 | 1 |
| 480 | thiol:disulfide interchange protein DsbG | 2 | 0 | 0 |
| 481 | thioredoxin family protein | 0 | 0 | 1 |
| 482 | Thiosulfate sulfurtransferase, rhodanese (EC 2.8.1.1) | 0 | 1 | 0 |
| 483 | thymidine phosphorylase family protein | 1 | 0 | 0 |
| 484 | Thymidylate synthase (EC 2.1.1.45) | 0 | 2 | 0 |
| 485 | TIGR00730 family Rossman fold protein | 1 | 0 | 0 |
| 486 | TIGR02391 family protein | 0 | 0 | 1 |
| 487 | TIGR03756 family integrating conjugative element protein | 1 | 0 | 0 |
| 488 | TIGR03757 family integrating conjugative element protein | 1 | 0 | 0 |
| 489 | TIGR03758 family integrating conjugative element protein | 2 | 0 | 0 |
| 490 | TIGR03759 family integrating conjugative element protein | 1 | 0 | 0 |
| 491 | TIGR03761 family integrating conjugative element protein | 2 | 0 | 0 |
| 492 | TIR domain-containing protein | 0 | 0 | 1 |
| 493 | TlpA family protein disulfide reductase | 2 | 0 | 1 |
| 494 | Tn4651 auxiliary cointegrate resolution protein T | 0 | 1 | 0 |
| 495 | TniQ family protein | 1 | 0 | 0 |
| 496 | TolC family protein | 2 | 0 | 0 |
| 497 | Toluene-4-monooxygenase, subunit TmoF | 0 | 1 | 0 |
| 498 | TraM recognition domain-containing protein | 1 | 0 | 0 |
| 499 | Transcription antitermination protein NusG | 0 | 1 | 0 |
| 500 | transcriptional regulator | 3 | 0 | 1 |
| 501 | transcriptional regulator MvaT, P16 subunit, putative | 0 | 2 | 0 |
| 502 | Transcriptional regulator PhnF | 0 | 1 | 0 |
| 503 | Transcriptional regulator YeiE, LysR family | 0 | 1 | 0 |
| **S. No.** | **Proteins encoded by sequences in genomic islands** | **APG1** | **APG2** | **APG4** |
| 504 | Transcriptional regulator, IclR family | 0 | 1 | 0 |
| 505 | Transcriptional regulator, MarR family | 0 | 1 | 0 |
| 506 | Transcriptional regulator, MecI family | 0 | 1 | 0 |
| 507 | transcriptional regulator, PbsX family | 0 | 1 | 0 |
| 508 | Transcriptional regulator, Xre family | 0 | 1 | 0 |
| 509 | transferase | 0 | 0 | 1 |
| 510 | Translation elongation factor G | 0 | 1 | 0 |
| 511 | Translation elongation factor Tu | 0 | 4 | 0 |
| 512 | translesion DNA synthesis-associated protein ImuA | 1 | 0 | 0 |
| 513 | translesion error-prone DNA polymerase V autoproteolytic subunit | 1 | 0 | 0 |
| 514 | transposase | 4 | 2 | 9 |
| 515 | Transposase and inactivated derivatives | 0 | 2 | 0 |
| 516 | TRAP dicarboxylate transporter, DctM subunit, unknown substrate 6 | 0 | 1 | 0 |
| 517 | TRAP dicarboxylate transporter, DctQ subunit, unknown substrate 6 | 0 | 1 | 0 |
| 518 | TRAP transporter solute receptor, unknown substrate 6 | 0 | 1 | 0 |
| 519 | TraR/DksA family transcriptional regulator | 1 | 0 | 0 |
| 520 | tRNA (cytidine(34)-2'-O)-methyltransferase (EC 2.1.1.207) | 0 | 1 | 0 |
| 521 | tRNA (uridine(34)/cytosine(34)/5- carboxymethylaminomethyluridine(34)-2'-O)- methyltransferase TrmL | 1 | 0 | 0 |
| 522 | tRNA(Cytosine32)-2-thiocytidine synthetase | 0 | 3 | 0 |
| 523 | two-component sensor histidine kinase | 1 | 0 | 1 |
| 524 | type I restriction endonuclease subunit R | 2 | 0 | 0 |
| 525 | type I restriction-modification system subunit M | 1 | 0 | 0 |
| 526 | Type I restriction-modification system, DNA-methyltransferase subunit M (EC 2.1.1.72) | 0 | 3 | 0 |
| 527 | Type I restriction-modification system, restriction subunit R (EC 3.1.21.3) | 0 | 2 | 0 |
| 528 | Type I restriction-modification system, specificity subunit S | 0 | 2 | 0 |
| 529 | type I secretion system permease/ATPase | 1 | 0 | 0 |
| 530 | type I toxin-antitoxin system SymE family toxin | 2 | 0 | 0 |
| 531 | type II glyceraldehyde-3-phosphate dehydrogenase | 0 | 0 | 1 |
| 532 | type II secretion system F family protein | 1 | 0 | 0 |
| 533 | type II secretion system major pseudopilin GspG | 1 | 0 | 0 |
| 534 | type II secretion system protein | 2 | 0 | 0 |
| 535 | type II toxin-antitoxin system RatA family toxin | 1 | 0 | 0 |
| 536 | type II toxin-antitoxin system VapC family toxin | 0 | 0 | 1 |
| 537 | Type III restriction-modification enzyme, helicase subunit | 0 | 1 | 0 |
| 538 | Type IV fimbrial biogenesis protein PilX | 0 | 1 | 0 |
| 539 | Type IV fimbrial biogenesis protein PilY1 | 0 | 1 | 0 |
| 540 | type IV pilin protein | 1 | 0 | 0 |
| 541 | Type IV pilus biogenesis protein PilE | 0 | 1 | 0 |
| 542 | type IV secretion system DNA-binding domain-containing protein | 1 | 0 | 0 |
| 543 | tyrosine-type recombinase/integrase | 0 | 0 | 2 |
| 544 | Tyrosyl-tRNA synthetase (EC 6.1.1.1) | 0 | 1 | 0 |
| 545 | UDP-2-acetamido-2-deoxy-D-glucuronic acid dehydrogenase (NAD+) (EC 1.1.1.335) | 0 | 1 | 0 |
| 546 | UDP-2-acetamido-3-amino-2,3-dideoxy-D- glucuronic acid acetyltransferase (EC 2.3.1.201) | 0 | 1 | 0 |
| 547 | UDP-glucose 4-epimerase (EC 5.1.3.2) | 0 | 1 | 0 |
| 548 | UDP-N-acetyl-D-glucosamine 6-dehydrogenase (EC 1.1.1.136) | 0 | 1 | 0 |
| 549 | Uncharacterized cysteine-rich DUF326 protein bsYhjQ/STM1261 | 0 | 2 | 0 |
| 550 | Uncharacterized MFS-type transporter | 0 | 1 | 0 |
| 551 | Uncharacterized Nudix hydrolase NudL | 0 | 1 | 0 |
| 552 | universal stress protein | 3 | 0 | 0 |
| 553 | UPF0225 protein YchJ | 0 | 1 | 0 |
| 554 | UPF0324 inner membrane protein YeiH | 0 | 1 | 0 |
| 555 | UPF0758 family protein | 0 | 1 | 0 |
| 556 | Urea ABC transporter, ATPase protein UrtD | 0 | 1 | 0 |
| 557 | Urea ABC transporter, ATPase protein UrtE | 0 | 1 | 0 |
| 558 | Urea ABC transporter, permease protein UrtB | 0 | 1 | 0 |
| 559 | Urea ABC transporter, permease protein UrtC | 0 | 1 | 0 |
| 560 | Urea ABC transporter, substrate binding protein UrtA | 0 | 1 | 0 |
| 561 | uridylate kinase | 2 | 0 | 0 |
| 562 | VacJ family lipoprotein | 1 | 0 | 0 |
| 563 | VanZ family protein | 0 | 0 | 1 |
| 564 | virulence RhuM family protein | 1 | 0 | 0 |
| 565 | VOC family protein | 0 | 0 | 1 |
| 566 | VRR-NUC domain-containing protein | 1 | 0 | 0 |
| 567 | WhiB family transcriptional regulator | 0 | 0 | 1 |
| 568 | winged helix-turn-helix transcriptional regulator | 0 | 0 | 2 |
| 569 | WYL domain-containing protein | 2 | 0 | 1 |
| 570 | XRE family transcriptional regulator | 1 | 0 | 0 |
| 571 | xylulose 5-phosphate 3-epimerase | 1 | 0 | 0 |
| 572 | y4eB gene in pNGR234a homolog | 0 | 1 | 0 |
| 573 | YbaB/EbfC family nucleoid-associated protein | 1 | 0 | 0 |
| 574 | YeeE/YedE family protein | 2 | 0 | 0 |
| 575 | Y-family DNA polymerase | 2 | 0 | 0 |
| **S. No.** | **Proteins encoded by sequences in genomic islands** | **APG1** | **APG2** | **APG4** |
| 576 | YHS domain-containing protein | 0 | 0 | 1 |
| 577 | YibE/F family protein | 0 | 0 | 1 |
| 578 | YnfA family protein | 0 | 0 | 1 |
| 579 | zinc metalloproteinase Mpr protein | 0 | 1 | 0 |
| 580 | zinc-ribbon domain-containing protein | 0 | 0 | 1 |
| 581 | hypothetical protein | 152 | 271 | 152 |
|  | Total | **506** | **591** | **378** |
