# Supplementary material for: Genome analysis to decipher syntrophy in the bacterial consortium ‘SCP’ for azo dye degradation
Source: BMC Microbiol. 2021 Jun 11;21:177. doi: 10.1186/s12866-021-02236-9 (PMC8194134; doi:10.1186/s12866-021-02236-9)
Supplement: Supplementary file 5 — Additional file 5. [file 12866_2021_2236_MOESM5_ESM.docx]

**Additional file 5: Figure S3.** Circular plots of APG genomes depicting genome islands as predicted by IslandPath-DIMOB and SIGI-HMM models in IslandViewer 4.
