# Supplementary material for: Genome analysis to decipher syntrophy in the bacterial consortium ‘SCP’ for azo dye degradation
Source: BMC Microbiol. 2021 Jun 11;21:177. doi: 10.1186/s12866-021-02236-9 (PMC8194134; doi:10.1186/s12866-021-02236-9)
Supplement: Supplementary file 6 — Additional file 6. [file 12866_2021_2236_MOESM6_ESM.docx]

**Additional file 6: Table S3.** (a) Insertion sequences identified in the genome of *Stenotrophomonas acidaminiphila* APG1 by ISfinder tool.

| **S. No.** | **IS elements** | **Family** | **Origin** | **Score (bits)** | **E-Value** |
| --- | --- | --- | --- | --- | --- |
| **1** | ISPa42 | Tn3 | *Pseudomonas aeruginosa* | 202 | 2.0E-49 |
| **2** | IS1389-B | IS3 | *Xanthomonas campestris* | 157 | 8.0E-36 |
| **3** | TnAs3 | Tn3Su | *Aeromonas salmonicidia* | 2416 | 0.0E+00 |
| **4** | SArsp9 | ISNCY | *Arthrobacter* sp. | 99.6 | 8.0E-19 |
| **5** | ISAzs17 | Tn3 | *Azospirillum* sp. | 52 | 1.0E-04 |
| **6** | ISVsp2 | IS1634 | *Verrucomicrobium spinosum* | 50.1 | 4.0E-04 |
| **7** | TnAs3 | Tn3 | *Aeromonas salmonicida* | 60 | 3.0E-07 |
| **8** | ISXoo13 | ISL3 | *Xanthomonas oryzae* | 101 | 9.0E-20 |
| **9** | ISAzs17 | Tn3 | *Azospirillum* sp*.* | 283 | 1.0E-74 |
| **10** | ISXoo13 | ISL3 | *Xanthomonas oryzae* | 101 | 8.0E-20 |
| **11** | ISXoo13 | ISL3 | *Xanthomonas oryzae* | 101 | 7.0E-20 |
| **12** | TnXo19 | Tn3 | *Xanthomonas oryzae* | 272 | 3.0E-71 |
| **13** | ISAav5 | IS481 | *Acidovorax avenae* | 75.8 | 4.0E-12 |
| **14** | TnAs3 | Tn3 | *Aeromonas salmonicida* | 60 | 2.0E-07 |
| **15** | ISXoo13 | ISL3 | *Xanthomonas oryzae* | 101 | 6.0E-20 |
| **16** | ISMno24 | IS91 | *Methylobacterium nodulans* | 56 | 3.0E-06 |
| **17** | ISStma13 | IS3 | *Stenotrophomonas maltophilia* | 97.6 | 9.0E-19 |
| **18** | TnAs3 | Tn3 | *Aeromonas salmonicida* | 63.9 | 1.0E-08 |
| **19** | ISPpn1 | IS21 | *Pandoraea pnomenusa* | 48.1 | 6.0E-04 |
| **20** | TnXo19 | Tn3 | *Xanthomonas oryzae* | 50.1 | 1.0E-04 |
| **21** | ISHwa21 | IS200/IS605 | *Haloquadratum walsbyi* | 48.1 | 2.0E-04 |
| **22** | ISHwa21 | IS200/IS605 | *Haloquadratum walsbyi* | 50.1 | 6.0E-05 |
| **23** | ISPye7 | IS5 | *Paracoccus yeei* | 52 | 7.0E-06 |
| **24** | ISXoo13 | ISL3 | *Xanthomonas oryzae* | 101 | 7.0E-21 |
| **25** | ISXoo13 | ISL3 | *Xanthomonas oryzae* | 101 | 6.0E-21 |
| **26** | TnAs3 | Tn3 | *Aeromonas salmonicida* | 44.1 | 8.0E-04 |
| **27** | ISMno11 | IS630 | *Methylobacterium nodulans* | 44.1 | 6.0E-04 |
| **28** | ISXoo13 | ISL3 | *Xanthomonas oryzae* | 260 | 5.0E-69 |

**Table S3 (b)** Insertion sequences identified in the genome of *Pseudomonas stutzeri* APG2 by ISfinder tool.

| **S. No.** | **IS elements** | **Family** | **Origin** | **Score (bits)** | **E-Value** |
| --- | --- | --- | --- | --- | --- |
| **1** | ISPsy43 | IS66 | *Pseudomonas syringae* | 75.8 | 3.0E-11 |
| **2** | ISPa40 | Tn3 | *Pseudomonas aeruginosa* | 2103 | 0.0E+00 |
| **3** | ISPst4 | IS3 | *Pseudomonas stutzeri* | 1132 | 0.0E+00 |
| **4** | ISPa61 | ISL3 | *Pseudomonas aeruginosa* | 194 | 5.0E-47 |
| **5** | ISPa61 | ISL3 | *Pseudomonas aeruginosa* | 190 | 7.0E-46 |
| **6** | ISPst4 | IS3 | *Pseudomonas stutzeri* | 186 | 1.0E-44 |
| **7** | ISPst4 | IS3 | *Pseudomonas stutzeri* | 196 | 1.0E-47 |
| **8** | ISPa86 | IS3 | *Pseudomonas aeruginosa* | 519 | 6.0E-145 |
| **9** | ISPa42 | Tn3 | *Pseudomonas aeruginosa* | 276 | 1.0E-71 |
| **10** | ISPst4 | IS3 | *Pseudomonas stutzeri* | 188 | 2.0E-45 |
| **11** | ISPst4 | IS3 | *Pseudomonas stutzeri* | 196 | 9.0E-48 |
| **12** | ISPst4 | IS3 | *Pseudomonas stutzeri* | 194 | 3.0E-47 |
| **13** | ISPa61 | ISL3 | *Pseudomonas aeruginosa* | 190 | 5.0E-46 |
| **14** | ISPst4 | IS3 | *Pseudomonas stutzeri* | 848 | 0.0E+00 |
| **15** | ISPa61 | ISL3 | *Pseudomonas aeruginosa* | 188 | 2.0E-45 |
| **16** | ISPa52 | IS5 | *Pseudomonas aeruginosa* | 323 | 4.0E-86 |
| **17** | ISPre1 | IS5 | *Pseudomonas resinovorans* | 569 | 4.0E-160 |
| **18** | ISPen2 | IS3 | *Pseudomonas entomophila* | 135 | 2.0E-29 |
| **19** | ISPst4 | IS3 | *Pseudomonas stutzeri* | 188 | 1.0E-45 |
| **20** | ISPst4 | IS3 | *Pseudomonas stutzeri* | 196 | 5.0E-48 |
| **21** | ISPa52 | IS5 | *Pseudomonas aeruginosa* | 323 | 3.0E-86 |
| **22** | ISPst3 | IS21 | *Pseudomonas stutzeri* | 3665 | 0.0E+00 |
| **23** | ISPsp6 | IS5 | *Pseudomonas* sp. | 196 | 3.0E-48 |
| **24** | ISPa52 | IS5 | *Pseudomonas aeruginosa* | 242 | 5.0E-62 |
| **25** | ISPst5 | IS5 | *Pseudomonas stutzeri* | 1465 | 0.0E+00 |
| **26** | ISPst5 | IS5 | *Pseudomonas stutzeri* | 1132 | 0.0E+00 |
| **27** | ISPsy42 | Tn3 | *Pseudomonas syringae* | 3051 | 0.0E+00 |
| **28** | ISPa61 | ISL3 | *Pseudomonas aeruginosa* | 194 | 5.0E-48 |
| **29** | ISPsy29 | IS3 | *Pseudomonas syringae* | 484 | 3.0E-135 |
| **30** | ISPa61 | ISL3 | *Pseudomonas aeruginosa* | 145 | 3.0E-33 |
| **31** | ISPst4 | IS3 | *Pseudomonas stutzeri* | 196 | 1.0E-48 |
| **32** | ISThsp9 | Tn3 | *Thiomonas* sp. | 248 | 3.0E-64 |
| **33** | ISCfr1 | IS1182 | *Citrobacter freundii* | 311 | 2.0E-83 |
| **34** | ISUnCu3 | IS21 | *Uncultured bacterium* | 466 | 4.0E-130 |
| **35** | ISPst4 | IS3 | *Pseudomonas stutzeri* | 188 | 1.0E-46 |
| **36** | ISPa61 | ISL3 | *Pseudomonas aeruginosa* | 188 | 9.0E-47 |
| **37** | ISPa52 | IS5 | *Pseudomonas aeruginosa* | 323 | 2.0E-87 |
| **38** | ISPpu22 | IS3 | *Pseudomonas putida* | 323 | 2.0E-87 |
| **39** | ISPa52 | IS5 | *Pseudomonas aeruginosa* | 234 | 1.0E-60 |
| **40** | TnAs1 | Tn3 | *Aeromonas salmonicida* | 54 | 2.0E-06 |
| **41** | ISPa52 | IS5 | *Pseudomonas aeruginosa* | 323 | 1.0E-87 |
| **42** | ISPst4 | IS3 | *Pseudomonas stutzeri* | 196 | 2.0E-49 |
| **43** | ISPa61 | ISL3 | *Pseudomonas aeruginosa* | 153 | 2.0E-36 |
| **44** | ISPa52 | IS5 | *Pseudomonas aeruginosa* | 234 | 6.0E-61 |
| **45** | ISPpu14 | IS66 | *Pseudomonas putida* | 242 | 2.0E-63 |
| **46** | ISPre1 | IS5 | *Pseudomonas resinovorans* | 892 | 0.0E+00 |
| **47** | IS881 | IS5 | *Ralstonia eutropha* | 936 | 0.0E+00 |
| **48** | ISPa52 | IS5 | *Pseudomonas aeruginosa* | 242 | 1.0E-63 |
| **49** | ISPa61 | ISL3 | *Pseudomonas aeruginosa* | 1879 | 0.0E+00 |
| **50** | ISPsp6 | IS5 | *Pseudomonas* sp. | 2391 | 0.0E+00 |

**Table S3 (c)** Insertion sequences identified in the genome of *Cellulomonas* sp. APG4 by ISfinder tool.

| **S. No.** | **IS elements** | **Family** | **Origin** | **Score (bits)** | **E-Value** |
| --- | --- | --- | --- | --- | --- |
| **1** | ISAcba1 | IS1595 | *Actinobacteria bacterium* | 244 | 1.0E-61 |
| **2** | ISAau2 | IS21 | *Arthrobacter aurescens* | 575 | 1.0E-161 |
| **3** | ISArsp6 | Tn3 | *Arthrobacter* sp. | 226 | 1.0E-56 |
| **4** | ISAcba1 | IS1595 | *Actinobacteria bacterium* | 65.9 | 2.0E-08 |
| **5** | ISBli29 | ISNCY | *Brevibacterium linens* | 517 | 2.0E-144 |
| **6** | ISAcba1 | IS1595 | *Actinobacteria bacterium* | 87.7 | 5.0E-15 |
| **7** | ISPfr2 | ISL3 | *Propionibacterium freudenreichii* | 105 | 2.0E-20 |
| **8** | ISAzs17 | Tn3 | *Azospirillum* sp. | 54 | 5.0E-05 |
| **9** | ISPfr12 | IS3 | *Propionibacterium freudenreichii* | 287 | 2.0E-75 |
| **10** | ISArsp6 | Tn3 | *Arthrobacter* sp. | 69.9 | 7.0E-10 |
| **11** | ISArsp14 | ISNCY | *Arthrobacter* sp. | 60 | 7.0E-07 |
| **12** | ISRae1 | IS481 | *Rhodococcus aetherivorans* | 167 | 4.0E-39 |
| **13** | ISAau2 | IS21 | *Arthrobacter aurescens* | 502 | 4.0E-140 |
| **14** | ISArsp9 | ISNCY | *Arthrobacter* sp. | 50.1 | 4.0E-04 |
| **15** | ISTesp1 | IS3 | *Terrabacter* sp. | 420 | 8.0E-116 |
| **16** | ISPsy43 | IS66 | *Pseudomonas syringae* | 58 | 1.0E-06 |
| **17** | ISPa43 | Tn3 | *Pseudomonas aeruginosa* | 50.1 | 2.0E-04 |
| **18** | ISPsy43 | IS66 | *Pseudomonas syringae* | 58 | 7.0E-07 |
| **19** | ISPfr2 | ISL3 | *Propionibacterium freudenreichii* | 103 | 7.0E-21 |
| **20** | ISAav1 | IS21 | *Acidovorax avenae* | 46.1 | 1.0E-03 |
| **21** | IS1096 | ISL3 | *Mycobacterium smegmatis* | 246 | 8.0E-65 |
