# Supplementary material for: Genome analysis to decipher syntrophy in the bacterial consortium ‘SCP’ for azo dye degradation
Source: BMC Microbiol. 2021 Jun 11;21:177. doi: 10.1186/s12866-021-02236-9 (PMC8194134; doi:10.1186/s12866-021-02236-9)
Supplement: Supplementary file 7 — Additional file 7. [file 12866_2021_2236_MOESM7_ESM.docx]

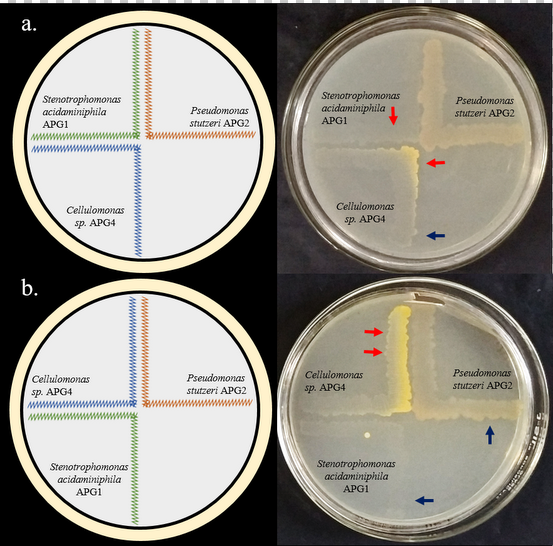


**Additional file 7: Figure S4.** Co-culturing of APG isolates on glycerol supplemented Bushnell Haas agar plates wherein (a) and (b) depict different orientations of the isolates. Red arrows represent the growth enhanced due to co-culturing, whereas the blue arrows indicate the growth of isolates independent of each other.
