# Supplementary material for: Genome analysis to decipher syntrophy in the bacterial consortium ‘SCP’ for azo dye degradation
Source: BMC Microbiol. 2021 Jun 11;21:177. doi: 10.1186/s12866-021-02236-9 (PMC8194134; doi:10.1186/s12866-021-02236-9)
Supplement: Supplementary file 8 — Additional file 8. [file 12866_2021_2236_MOESM8_ESM.docx]

**Additional file 8: Table S4.** Genetic composition of the three organisms concerning glycerol dissimilation.

| **Pathway** | **Enzymes involved in glycerol utilization** | **APG1** | **APG2** | **APG4** |
| --- | --- | --- | --- | --- |
| Uptake of glycerol | Glycerol uptake facilitator protein, GlpF | 0 | 1 | 1 |
| Glycerol degradation I (Phosphorylative pathway) | Glycerol kinase (EC 2.7.1.30) | 0 | 1 | 1 |
|  | Aerobic glycerol-3-phosphate dehydrogenase (EC 1.1.5.3) | 0 | 2 | 1 |
|  | DeoR family transcriptional regulator, GlpR | 0 | 1 | 1 |
| Glycerol degradation pathway II  (Oxidative pathway) | Dihydroxyacetone kinase, ATP-dependent (EC 2.7.1.29) | 0 | 0 | 2 |
|  | Glycerol dehydrogenase, NAD-dependent (EC 1.1.1.6) | 0 | 0 | 0 |
| Glycerol degradation pathway III  (Reductive pathway) | Glycerol dehydratase (EC 4.2.1.30) | 0 | 0 | 0 |
|  | 1,3-propanediol dehydrogenase (EC 1.1.1.202) | 0 | 0 | 0 |
| Glycerol degradation V | Glycerol dehydrogenase, NAD-dependent (EC 1.1.1.6) | 0 | 0 | 0 |
|  | Dihydroxyacetone kinase, PEP-dependent (EC 2.7.1.121) | 0 | 0 | 3 |
| Glycerol and glycerophosphodiester degradation | Glycerophosphoryl diester phosphodiesterase (EC 3.1.4.46) | 1 | 2 | 1 |
|  | Aerobic glycerol-3-phosphate dehydrogenase (EC 1.1.5.3) | 0 | 2 | 1 |
|  | Glycerol-3-phosphate transporter, GlpT | 0 | 0 | 0 |
