# Supplementary material for: Genome analysis to decipher syntrophy in the bacterial consortium ‘SCP’ for azo dye degradation
Source: BMC Microbiol. 2021 Jun 11;21:177. doi: 10.1186/s12866-021-02236-9 (PMC8194134; doi:10.1186/s12866-021-02236-9)
Supplement: Supplementary file 9 — Additional file 9. [file 12866_2021_2236_MOESM9_ESM.docx]

**Additional file 9: Table S5.** Comparison of the enzymes from all the genomes probably involved in RB28 degradation.

| **S. No.** | **Enzyme** | **EC** | **Number of ORFs** | | |
| --- | --- | --- | --- | --- | --- |
|  |  |  | **APG1** | **APG2** | **APG4** |
|  | **Enzymes active in reductive degradation** |  |  |  |  |
| **E1** | FMN-dependent NADH-azoreductase | 1.7.1.6 | 0 | 1 | 1 |
| **E2** | NADH dehydrogenase oxidoreductase (chain A-N) | 1.6.5.3 | 1 | 0 | 1 |
| **E3** | NADH dehydrogenase/NADH:DCIP oxidoreductase | 1.6.99.3 | 1 | 1 | 3 |
| **E4** | Sulfatase | 3.1.6.- | 1 | 0 | 0 |
|  | **Enzymes active in oxidative degradation** |  |  |  |  |
| **E5** | Laccase (AA1) | 1.10.3.2 | 0 | 0 | 0 |
| **E6** | Manganese peroxidase, MnP (AA2) | 1.11.1.13 | 0 | 0 | 0 |
| **E7** | Cytochrome P450 | - | 0 | 0 | 1 |
| **E8** | Lignin peroxidase, LiP (AA2) | 1.11.1.14 | 0 | 0 | 0 |
| **E9** | Peroxidases (AA2) | 1.11.1.- | 0 | 0 | 0 |
| **E10** | Versatile peroxidase (AA2) | 1.11.1.16 | 0 | 0 | 0 |
| **E11** | Quinone-dependent oxidoreductase (AA12) | 1.-.-.- | 0 | 1 | 0 |
| **E12** | Tyrosinase, Tyr | 1.14.18.1 | 0 | 0 | 0 |
| **E13** | Lytic polysaccharide monooxygenases, LPMOs (AA12) | - | 0 | 0 | 0 |
|  | **H_2_O_2_ producing enzymes** |  |  |  |  |
| **E14** | Glucose 1-oxidase (AA3) | 1.1.3.4 | 0 | 0 | 0 |
| **E15** | Alcohol oxidase (AA3) | 1.1.3.13 | 0 | 0 | 0 |
| **E16** | Cellobiose dehydrogenase (AA3) | 1.1.99.18 | 0 | 0 | 0 |
| **E17** | Vanillyl-alcohol oxidase (AA4) | 1.1.3.38 | 1 | 0 | 0 |
| **E18** | Galactose oxidase (AA5) | 1.1.3.9 | 0 | 0 | 0 |
| **E19** | Glyoxal oxidase (AA5) | 1.2.3.15 | 0 | 0 | 0 |
| **E20** | Aryl alcohol oxidase (AA3) | 1.1.3.7 | 0 | 0 | 0 |
| **E21** | Pyranose oxidase (AA3) | 1.1.3.10 | 0 | 0 | 0 |
|  | **Electron transport enzymes** |  |  |  |  |
|  | Quinone reductases |  |  |  |  |
| **E22** | NADPH:Quinone oxidoreductase | 1.6.5.5 | 0 | 1 | 2 |
| **E23** | 1,4-benzoquinone reductase (AA6) | 1.6.5.6 | 1 | 2 | 1 |
|  | Riboflavin-related enzymes |  |  |  |  |
| **E24** | Riboflavin synthase eubacterial/eukaryotic | 2.5.1.9 | 1 | 1 | 1 |
| **E25** | FMN adenylyltransferase /Riboflavin kinase | 2.7.7.2 / 2.7.1.26 | 1 | 1 | 1 |
| **E26** | Riboflavin transporter PnuX | - | 0 | 0 | 1 |
|  | Others |  |  |  |  |
| **E27** | Phenazine biosynthesis protein PhzF like | 5.3.3.17 | 2 | 1 | 1 |
| **E28** | Cobalamin synthase | 2.7.8.26 | 1 | 1 | 0 |
|  | **Membrane-bound components (not included in Fig 6)** |  |  |  |  |
|  | Cytochrome c oxidase |  |  |  |  |
| **1** | Cytochrome c oxidase polypeptide I | 1.9.3.1 | 2 | 4 | 1 |
| **2** | Cytochrome c oxidase polypeptide II | 1.9.3.2 | 1 | 4 | 1 |
| **3** | Cytochrome c oxidase polypeptide III | 1.9.3.3 | 1 | 4 | 1 |
| **4** | Cytochrome c oxidase polypeptide IV | 1.9.3.4 | 0 | 0 | 1 |
| **5** | Cytochrome c oxidase (cbb3-type) subunit CcoO | 1.9.3.1 | 0 | 3 | 0 |
| **6** | Cytochrome c oxidase (cbb3-type) subunit CcoN | 1.9.3.1 | 1 | 3 | 0 |
| **7** | Cytochrome c oxidase (cbb3-type) subunit CcoP | 1.9.3.2 | 1 | 2 | 0 |
| 8 | Cytochrome c oxidase (cbb3-type) subunit CcoQ | 1.9.3.3 | 1 | 1 | 0 |
