# Supplementary material for: Genome analysis to decipher syntrophy in the bacterial consortium ‘SCP’ for azo dye degradation
Source: BMC Microbiol. 2021 Jun 11;21:177. doi: 10.1186/s12866-021-02236-9 (PMC8194134; doi:10.1186/s12866-021-02236-9)
Supplement: Supplementary file 11 — Additional file 11. [file 12866_2021_2236_MOESM11_ESM.docx]

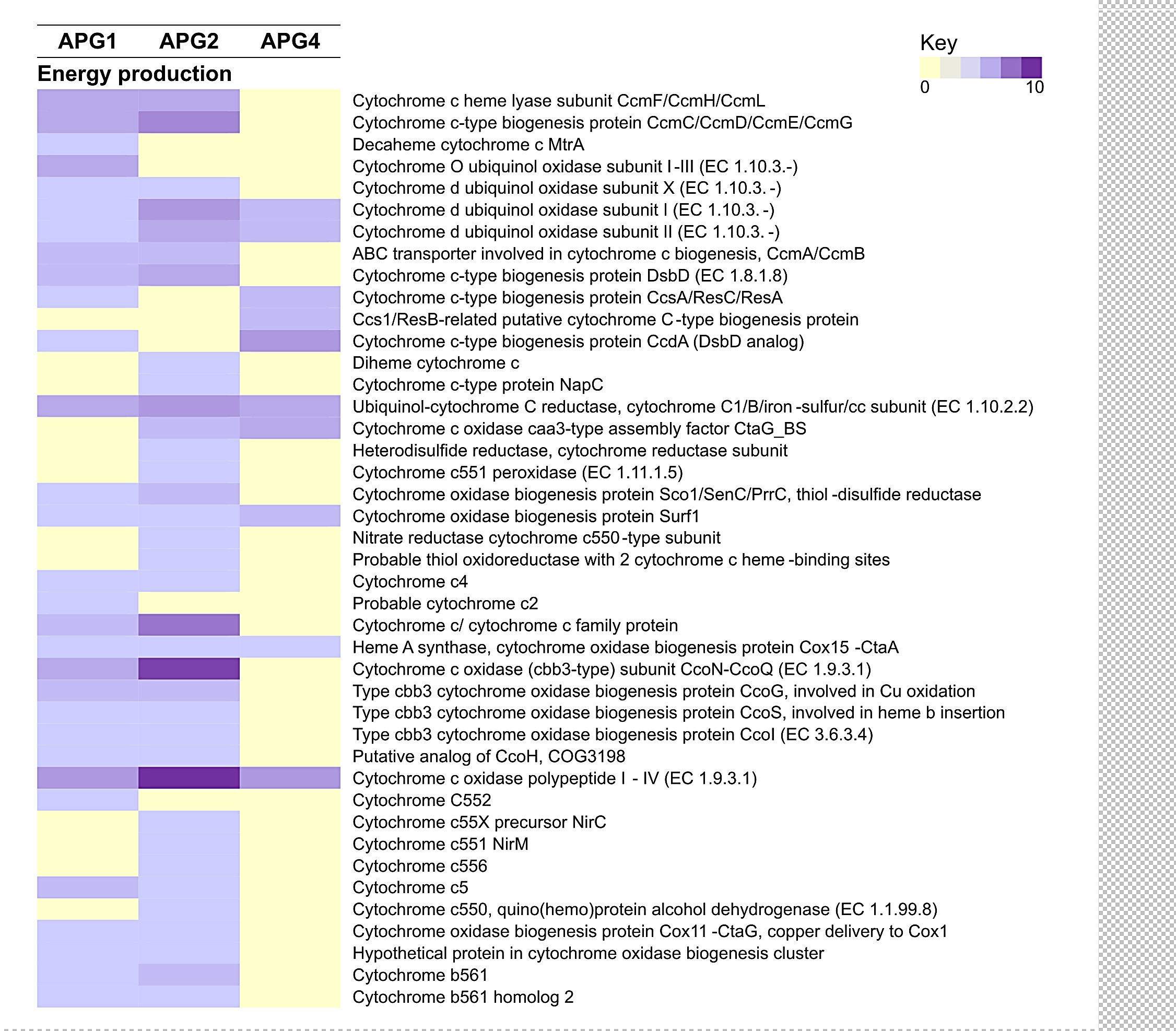


**Additional file 11: Figure S6.** Genes dedicated to energy production and conservation in APG genomes.
