# Supplementary material for: Genome analysis to decipher syntrophy in the bacterial consortium ‘SCP’ for azo dye degradation
Source: BMC Microbiol. 2021 Jun 11;21:177. doi: 10.1186/s12866-021-02236-9 (PMC8194134; doi:10.1186/s12866-021-02236-9)
Supplement: Supplementary file 12 — Additional file 12. [file 12866_2021_2236_MOESM12_ESM.docx]

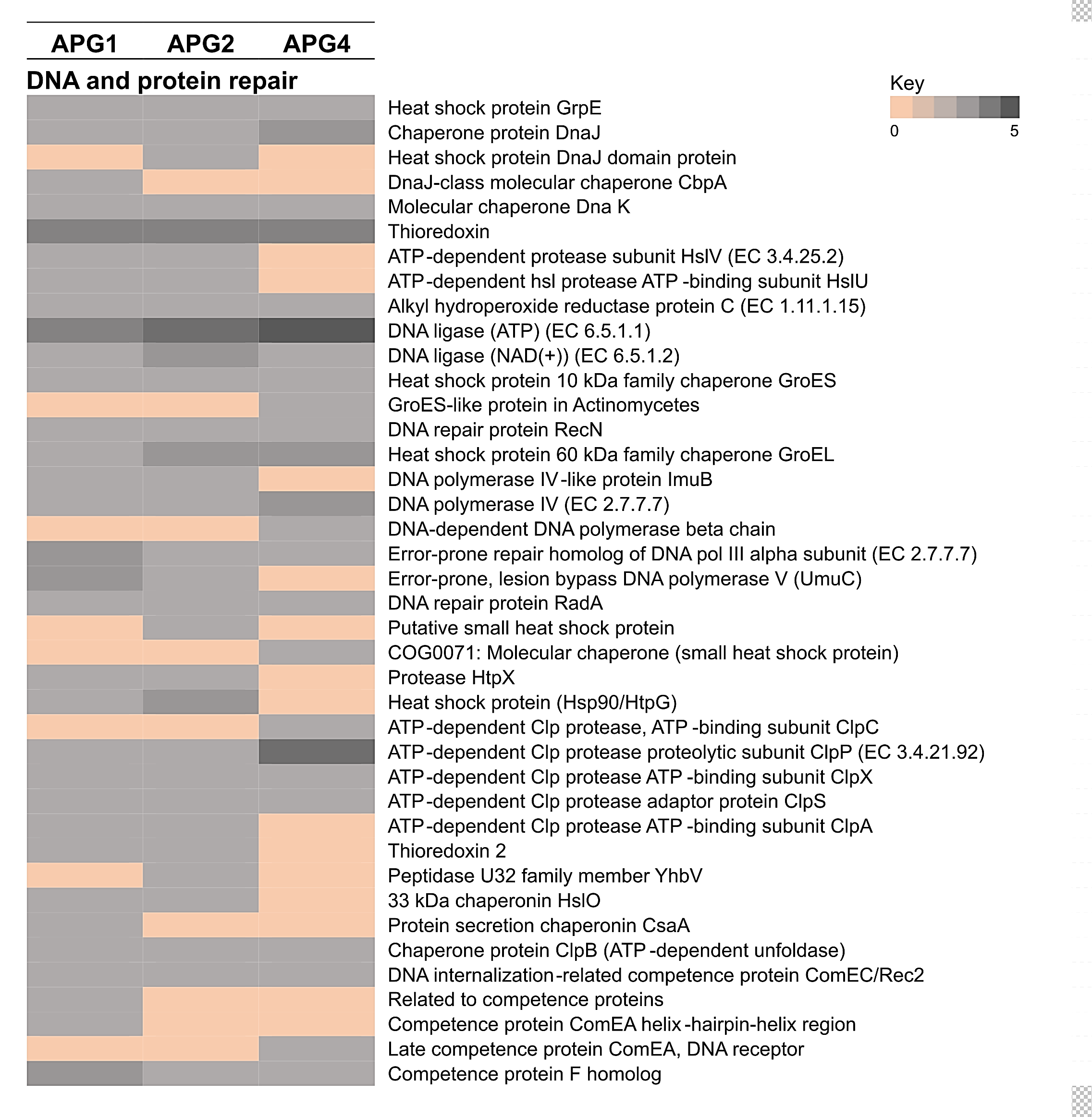


**Additional file 12: Figure S7.** Genes of APG genomes associated with DNA and protein repair during degradation.
