# Supplementary figures and images for: Genome analysis to decipher syntrophy in the bacterial consortium ‘SCP’ for azo dye degradation
Source: BMC Microbiol. 2021 Jun 11;21:177. doi: 10.1186/s12866-021-02236-9 (PMC8194134; doi:10.1186/s12866-021-02236-9)

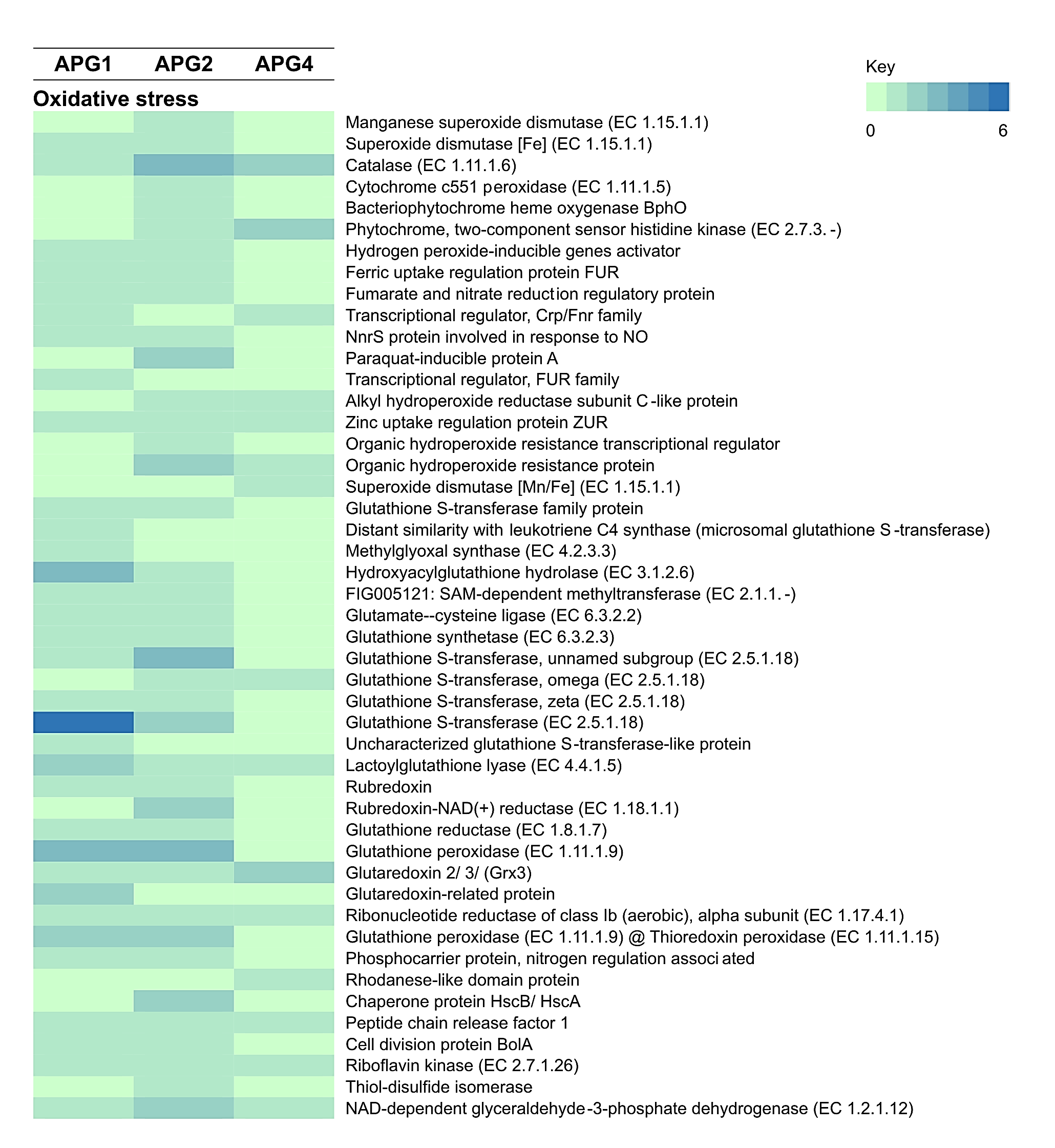


**Additional file 13: Figure S8.** Genes encoding for proteins involved in combating oxidative stress.

Supplement: Supplementary file 13 — Additional file 13. [file 12866_2021_2236_MOESM13_ESM.docx]

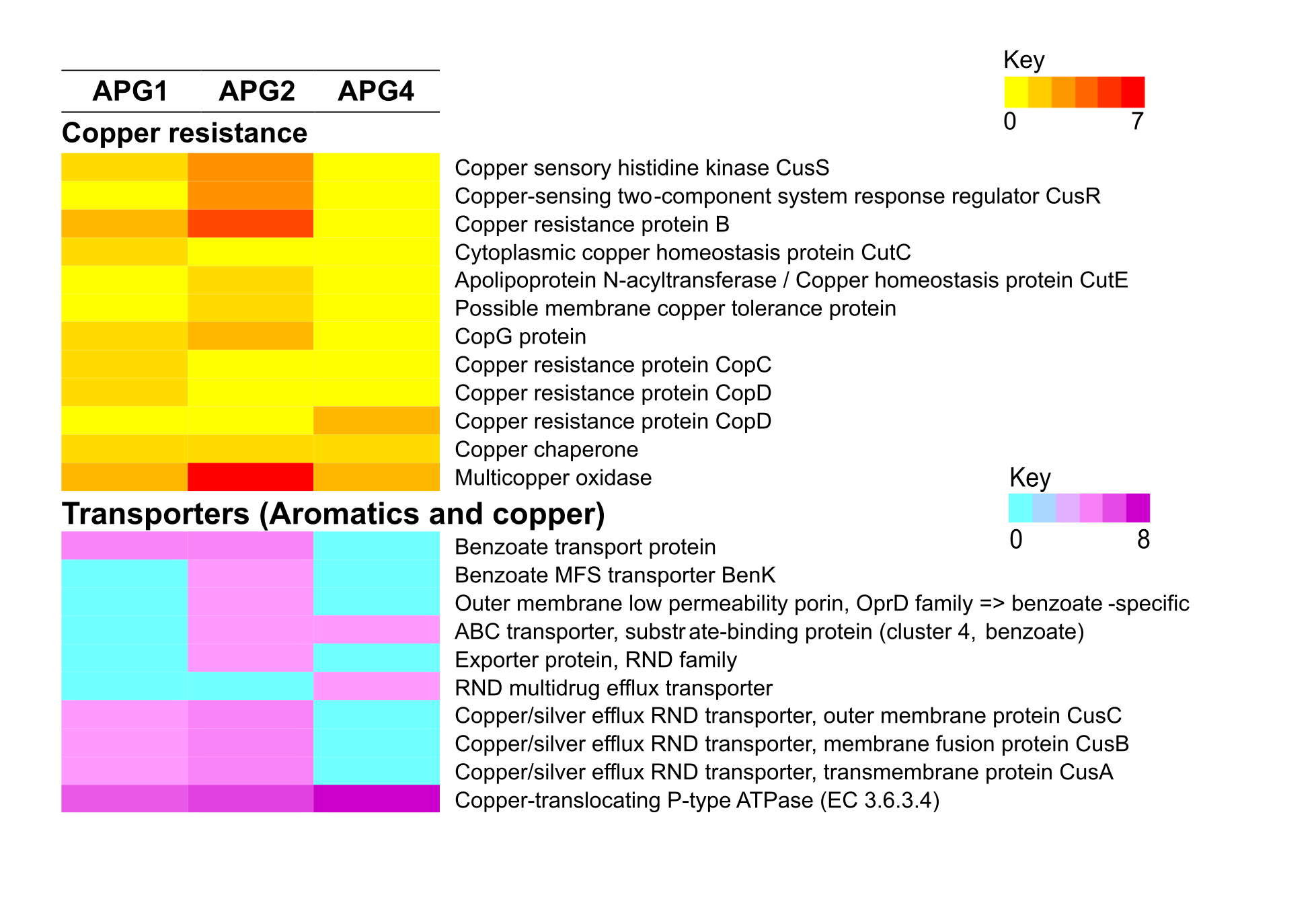


**Additional file 15: Figure S10.** Genes of APG genomes involved in copper resistance and transport.

Supplement: Supplementary file 15 — Additional file 15. [file 12866_2021_2236_MOESM15_ESM.docx]
